# Mineral medicine: from traditional drugs to multifunctional delivery systems

**DOI:** 10.1186/s13020-022-00577-9

**Published:** 2022-02-10

**Authors:** Xiaoqing Zhong, Zhenning Di, Yuanxin Xu, Qifan Liang, Kuanhan Feng, Yuting Zhang, Liuqing Di, Ruoning Wang

**Affiliations:** 1 College of Pharmacy, Nanjing University of Chinese Medicine, Nanjing, 210023 China; 2 Jiangsu Provincial TCM Engineering Technology Research Center of High Efficient Drug Delivery System, Nanjing, 210023 China

**Keywords:** Mineral drug, Traditional preparation, Novel drug delivery system, Active ingredient

## Abstract

Mineral drugs are an important constituent of traditional Chinese medicine (TCM). Taking minerals that contain heavy metals as drugs is a very national characteristic part of TCM. However, the safety and scientific nature of mineral drugs are controversial owing to their heavy metals and strong toxicity. In 2000, the Food and Drug Administration (FDA) authorized arsenic trioxide (ATO) as first-line therapy for acute promyelocytic leukemia. This makes the development and utilization of mineral drugs become a research hotspot. The development of nanomedicine has found a great prospect of mineral drugs in nano-delivery carriers. And that will hold promise to address the numerous biological barriers facing mineral drug formulations. However, the studies on mineral drugs in the delivery system are few at present. There is also a lack of a detailed description of mineral drug delivery systems. In this review, the advanced strategies of mineral drug delivery systems in tumor therapy are summarized. In addition, the therapeutic advantages and research progress of novel mineral drug delivery systems are also discussed. Here, we hope that this will provide a useful reference for the design and application of new mineral drug delivery systems.

**Graphical Abstract:**

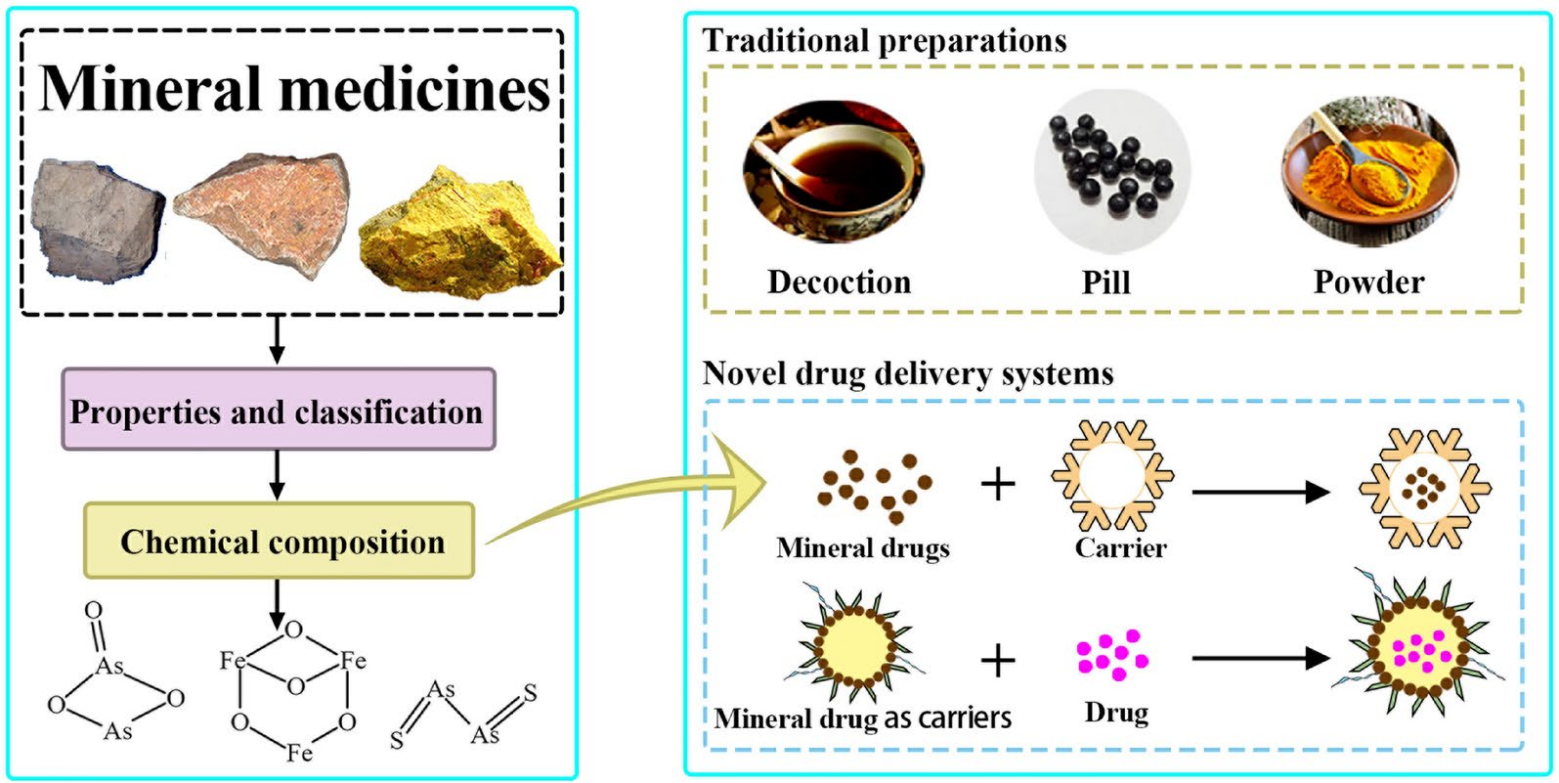

## Background

Minerals are rich in resources in China and have significant curative effects as medicines, and are a unique component of traditional Chinese medicine (TCM). The research and utilization of mineral drug resources have a history of more than 2000 years. As early as the Spring–autumn Warring period, there were two kinds of mineral drugs used to treat diseases among the 122 kinds of drugs. Fifty-two disease prescriptions, the earliest existing medical classic, recorded the clinical application of 20 kinds of mineral drugs, such as realgar, cinnabar, and saltpeter. With the in-depth study of TCM, mineral medicine has gradually become a major research hotspot of drug development and application at home and abroad. Mineral medicine is a general term for natural minerals with medicinal value, mineral processed products and fossils of animals or animal bones. Among them, natural minerals such as cinnabar, gypsum, calamine, and ochre. The processed products of minerals include light powder, red powder, autumn stone and so on. Fossils of animals or animal bones include keels, stone swallows, etc. [1–3]. Modern pharmacology found that many mineral drugs have a good curative effect for a variety of malignant tumors, such as arsenic and cinnabar. Yet, the practical trial of mineral drugs is curbed on account of the problems of inferior water solubility, high content of heavy metals, and severe toxicity. Recently, the research of some novel drug delivery techniques has brought opportunities for the development and adoption of mineral drugs.

**Fig. 1:**
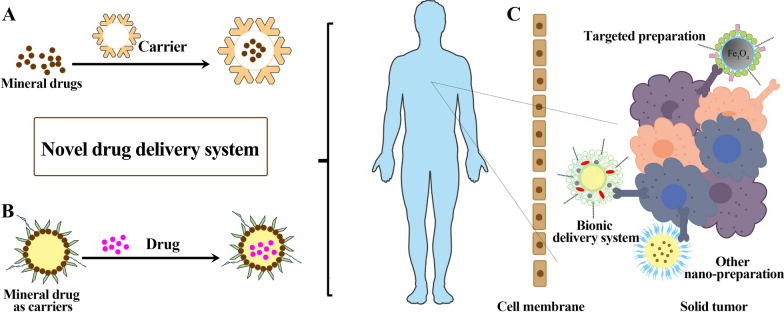
Schematic diagram of the role that mineral medicines play in modern drug delivery systems. Mineral drugs can function in novel delivery systems as synergistic drugs (**A**) and drug delivery vehicles (**B**), respectively. **C** Mineral drug nanoparticles can penetrate cell membranes and precisely target tumor cells

Many studies focus on the drug delivery systems loaded mineral drugs, to acquire advanced efficacy and decreased toxicity with less adverse reactions. As shown in Fig. 1A, B, mineral drugs usually play a significant role in tumor therapy in the forms of drugs or carriers. Figure 1C shows that mineral drug nanoparticles can accurately penetrate the cell membrane to the tumor tissues. Ettlinger et al. designed a kind of zeolitic imidazolate skeleton-8 as the pH-responsive messenger of arsenic trioxide (ATO), which has high remedy loading and significant pH triggered release behavior at the same time [1]. It has been proved to be a promising candidate material for transporting arsenic compounds. According to literature reports polylactic acid/magnetic compound nano-particles, liposome complexes, amino acid, and other materials are used to deliver ATO to revamp the cure of arsenate in carcinoma treatment [2–4]. Another typical mineral drug is magnetite, which is mainly composed of Fe_3_O_4_. Rayegan et al. loaded cephalexin (CPX) on magnetic nanoparticles encapsulated in basilicum seed mucilage (BSM) to prepare the complex with more gradual and sustained drug release (Fe_3_O_4_@BSM-CPX). With the help of magnetic targeting of Fe_3_O_4_, a further drug send system was developed, and its antibacterial ability of the complex was significantly increased [5].

This review systematically summarizes the latest research on the enhancement of the curative effect of mineral drugs in the drug delivery system. Of note, the classification and active components of mineral drugs are introduced to promote the understanding of mineral drugs. More importantly, the modern pharmacological effects of mineral drugs are described in detail. Based on the active components and pharmacological activities of mineral drugs, the recent research progress of mineral drugs combined with novel drug delivery systems is highlighted. In the end, some critical challenges in the research of mineral drugs based on drug loading systems are discussed to provide a reference for the future development of mineral medicines.

### Properties and classification of Chinese mineral medicine

Mineral drugs of TCM include original minerals (cinnabar, gypsum, calamine, etc.), processed products with minerals as raw materials (calomel, mirabilite, etc.), fossils of animals, or animal bones (fossil fragments, dragon teeth, etc.). It is a part of the characteristics of Chinese medicine and has an idiographic function in the progress of TCM [6]. With the development of the internationalization of TCM, the research of traditional mineral medicines is gradually in-depth and extensive. In this section, we will outline the properties and classification of mineral drugs.

### Mineral properties of Chinese medicine

**Fig. 2:**
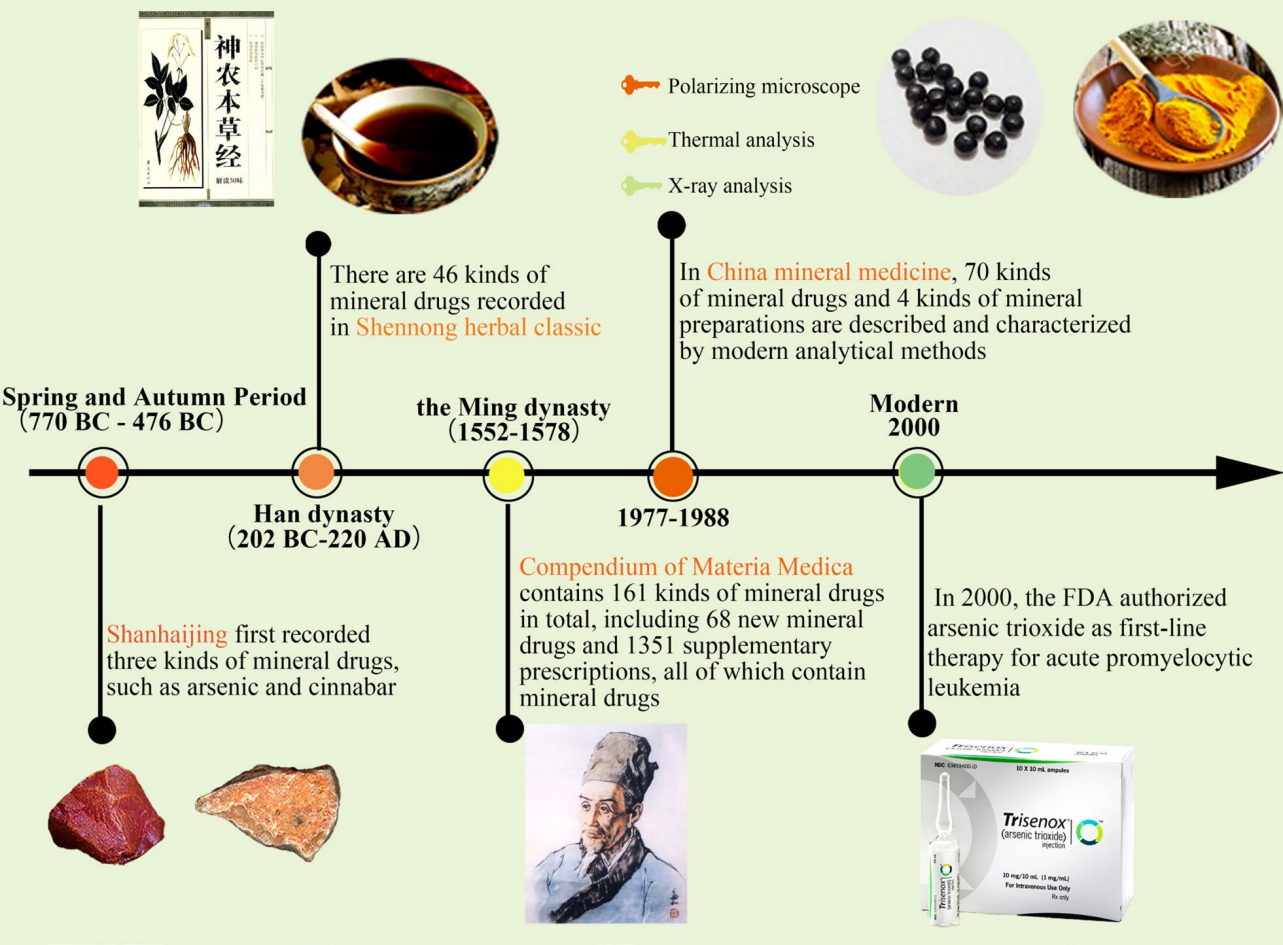
Illustration of the development process of mineral pharmaceutical preparations. The application of mineral medicine was first recorded in “Shanhaijing”. “Shennong herbal classic” and “Compendium of Materia Medica” have sorted out the mineral medicines. In the twenty-first century, with the development of modern scientific and technological means, people's understanding of mineral drugs has gradually deepened. The FDA's first approval of arsenic trioxide as a first-line anticancer drug is of epoch-making significance. (The copyright of the pictures from network sources in the figure is shown below. Copyright^©^ 2012–2021 Chengdu Gaoxin bingzhengtang TCM outpatient department Co., Ltd. all rights reserved. Copyright@2014 www.fl2j.com. Copyright^©^ 2013 Phoenix New Media Limited All Rights Reserved. Copyright^©^ 2019 www.tcmdoc.cn. Copyright^©^ 2008–2013 gucn.com. Copyright^©^ 2008 https://www.zhihu.com/. Copyright^©^ https://www.hrhnyy.cn/. Copyright 1996–2006 Cerner Multum, Inc.)

Medicate with minerals, especially mineral medicine containing heavy metals, is a very extraordinary part of TCM. In the second century before Christ, mercury could be refined from cinnabar. In Song Dynasty, there are 139 kinds recorded in Zheng Lei Ben Cao and 222 kinds recruited in the Compendium of Materia Medica. Mineral drugs are abundant in resources and used widely. Figure 2 vividly shows the development of mineral medicine for thousands of years, indicating that mineral medicine has always been a part of TCM.

Except for a few natural elements, mineral drugs are mostly composed of a lot of compounds. Each mineral has certain physical and chemical properties. The knowledge of mineral medicine is generally identified from its appearance, physical and chemical properties, and microscopic observation. The appearance properties of mineral medicine include color, streaks, texture, smell, hardness, cleavage, fracture, magnetism, and specific gravity. Of note, the pyrite is dense, massive, and has green–black or red–brown streaks. Meanwhile, magnetite, commonly used in Chinese medicine for tranquillization, is gray–black, irregular block, metallic luster. Chlorite schist is greenish-black with glassy luster, and its cross-section is lamellar. Among them, mineral medicine has its inherent streak color. The streak color is the color of the powder trace left by the mineral after scratching on the white wool porcelain plate. The streak color may be different from the color of the mineral itself. For example, the surface of the pyritum is bright yellow, and the streak color is green–black to brown–red. In addition, microscopic identification can more accurately identify drugs from the optical properties of mineral drugs. At present, the identification of mineral medicines is gradually inclined to rely on various modern scientific and technological means, such as X-ray diffraction, electron microscopy, atomic absorption spectroscopy, thermal analysis, phase analysis, infrared spectroscopy, and nuclear magnetic resonance.

In recent years, the pharmacodynamic research of traditional Chinese medicine decoction has become more and more in-depth. Some studies believe that the metal ions in mineral medicines may participate in the formation of nanostructures of other ingredients in the decoction through coordination, forming self-assembled nanoparticles, thus exerting their medicinal effects. With the expansion of mineral drug research, many mineral nanoparticles have attracted extensive attention in the fields of bioengineering, biomedicine, and food analysis because of their unique properties [7]. For example, Fe_3_O_4_ nanoparticles have the properties of superparamagnetic, high external area, and low toxicity, which are widely used to enhance medicament efficacy and improve cancer treatment effects in the magnetic targeted delivery systems [8, 9]. Table 1 summarizes the classification and efficacy indications of mineral drugs in TCM.

**Table 1:** Summary of active components and pharmacological effects of mineral drugs

Compound	Source	Efficacy indications	Pharmacological activity and mechanism
Mercury	Cinnabar (HgS)	Tranquillization with heavy prescription, improving eyesight, and removing toxicity substances	Conserve dopaminergic neurons from lipopolysaccharide-induced neurotoxicity by suppressing the microglial activation and proinflammatory factor production [114] Neuroprotective effect on ischemic stroke and reperfusion injury [115] Neuroprotective effect on neurodegenerative diseases [116] Reduce oxidative stress by regulating 5-HT metabolism [126]
Iron	Magnetite (Fe_3_O_4)_	Tranquillization with heavy prescription and suppressing hyperactive liver and subsiding yang	Induce apoptosis of A549 lung adenocarcinoma cell line [127]
	Ochre (Fe_2_O_3)_	Suppressing hyperactive liver for descending adverse qi and cooling blood for hemostasis	Inhibit the expression of some inflammatory cytokines and interferon response genes, regulate T cell immune activity, and play an anti-inflammatory role [128] Induction of TNF in RAW264.7 cells- α and IL-6 expression [129, 130]
Calcium	Gypsum (CaSO_4_·2H_2_O)	Clearing heat-fire and quenching thirst	Increase the utterance level of Aquaporin-3, accelerate the formation of collagen and capillaries on the wound, and promote the proliferation of granulation tissue, thereby promoting the healing of the skin wound [131, 132]
Arsenic	Realgar (As_2_S_2_)	Killing ascarid and removing toxic substances, eliminating dampness and phlegm, and preventing malaria	Inhibit the proliferation of leukemia cell line F-36P and induce its apoptosis [133] Inducing apoptosis of human liver cancer HepG2, QGY-7703 cells and blocking cell cycle G2/M progression [134–137] Inhibit the overexpression of microRNA-372 to restrain the pervasion and diaspora of prostate neoplasm cells [138] Prohibition of gastric carcinoma cells metastasis via the NFA Tc3/c-Myc roadway [139, 140] Repress the activity of MCF-7 and MDA-MB-231 in human chest cancer cells [91, 141]
	Arsenic trioxide (As_2_O_3_)	Eliminating pus and necrotic tissues, dissipating phlegm, and preventing malaria	Induce Molt-4 and Mutz-1 autophagy damage in leukemia cells [133]
Aluminum	Alum (KAl (SO_4_)_2_·12H_2_O)	Killing ascarid and removing toxicity substance for external, eliminating phlegm for calming endogenous wind and hemostasis for internal	Reduce the content of collagen in the myometrium, gene representation, and albumen concentration of MMP-2 and MMP-9 to improve uterine leiomyoma [142]
	Halloysite (Al_4_(Si_4_O_10_) (OH)_8_ · 4H_2_O)	Astringing sores, promoting granulation and hemostasis	Protect locally inflamed gastrointestinal mucosa
Copper	Blue vitriol (CuSO_4_·5H_2_O)	Eliminating necrosis and detoxication	Promote bile secretion and have a sialagogic effect
Sodium	Mirabilite (Na_2_SO_4_·10H_2_O)	Clearing heat for detumescence and moistening dryness for relaxing bowels	Promote intestinal activity and improve gastrointestinal dysfunction [143]
Magnesium	Talc (Mg_3_(Si_4_O_10_) (OH)_2)_	Clearing heat and freeing strangury	Protect the inflamed gastrointestinal mucosa or damaged skin tissues [144]

### Common classification of mineral drugs

In traditional Chinese medicine theory, mineral medicines can be divided into five categories according to their properties and functions: sedation and tranquility, tourniquet hemostasis, detoxification, vigorous purification of drainage, and insecticide. For example, cinnabar and magnetite have a sedative effect, and ochre has a hemostatic effect. At present, the modern classification of mineral medicines is mostly classified according to the type of anion or cation that is the main component of the mineral. The Chinese Pharmacopoeia adopts anion classification. For example, cinnabar and realgar are sulfur compounds, gypsum and strange stone are sulfates, calcium amines are carbonates, magnetite and ochre are oxides, and calcium amines are halides. Cation classifications include mercury compounds, arsenic compounds, lead compounds, iron compounds, calcium compounds, aluminum compounds, copper compounds, sodium compounds, and magnesium compounds [4, 5].

### Chemical constituents of minerals in traditional Chinese medicine

Most mineral drugs are compounds containing heavy metal elements. The main ingredients include arsenic, lead, mercury, copper, iron, magnesium, aluminum, calcium, silicate, sulfate, and other ingredients. These ingredients include trace elements required by the human body and have an impact on human health, which has attracted wide attention from researchers.

### Chemical composition and pharmacological action of mineral medicine

Many mineral drugs have significant pharmacological activity. Copper, iron, etc. are common metal elements and trace elements necessary for the human body. Lack of it can cause physiological dysfunction and cause disease. Copper has certain effects on cardiovascular, bone marrow, central nervous system, and red blood cell production. Copper participates in the body’s biochemical functions such as oxidative phosphorylation, free radical detoxification, melanin synthesis, catecholamine metabolism, connective tissue cross-linking, blood coagulation, and hair formation. Azurite ore, chlorocopper ore, and bornite ore can all be used as medicine. CuSO_4_·5H_2_O is the main component of chalcogenide and has a good antibacterial effect. Studies have shown that, compared with ordinary copper oxide, nano-copper oxide has superior properties such as surface effect, quantum size effect, volume effect, and macroscopic quantum tunneling effect [10, 11]. Iron is the most abundant and important hematopoietic element in the human body. Iron-containing drugs mainly exist as iron elements, iron oxide, iron sulfide, or iron sulfate. Mineral drugs containing iron and its compounds are clinically used to treat convulsions, swelling toxins, iron-deficiency anemia, and various causes of blood deficiency and blood deficiency. Among them, magnetite mainly contains iron tetroxide, ochre mainly contains iron trioxide, and pyrite mainly contains iron disulfide. With the research of nanomedicine, magnetic nanoparticles have shown positive advantages in biomedicine and drug delivery [12–15].

Calcium and sodium are also common mineral medicine ingredients. Calcium-containing mineral medicines mainly contain oxides, carbonates, and calcium fluoride as their main active ingredients. Calcium-containing mineral drugs have a wide range of clinical applications in gynecological diseases. In addition, calcium mineral medicine also has a special anti-convulsant and soothing effect and is clinically used for epileptic mania. For example, the main component of amethyst is CaF_2_, which can excite the central nervous system and ovarian secretion function. Sodium-containing mineral drugs are mainly used clinically to reduce local leukocyte infiltration, regulate body immunity, and prevent postoperative infections. For example, borax mainly contains sodium tetraborate, which has antiseptic and anticonvulsant effects. Glauber's salt mainly contains water-containing sodium sulfate, which has anti-inflammatory, anti-bacterial, and laxative effects.

Mineral medicine also contains non-metallic compounds, such as talc, white quartz, and other traditional Chinese medicines containing silicon. Talc is a silicate mineral, mainly containing hydrous magnesium silicate, which has the function of protecting skin and mucous membranes and antibacterial. The main component of white quartz is silica, which has sedative, calming, antitussive and anti-asthmatic effects. In addition, silica, the main component of the quartz photo album, can be used as a load-bearing coating for various nano-drugs, exaggerating the stability of the nano-particles and increasing the efficacy of the nano-particles [16–19].

### Toxicity of mineral medicine

Some toxic heavy metals also have medicinal value, such as mineral medicines containing mercury, lead, gold, and silver. For example, cinnabar is a natural ore of mercury sulfide. HgS is a substance that is hardly soluble in water and organic solvents. Under the action of acid effect and complexing effect in the human body, it can dissociate and complex reaction with amino acid and other biological molecules to form complexes with strong physiological activity and less toxicity. Therefore, cinnabar can act on the central nervous system, with hypnotic, brain protection, anti-fear, anti-anxiety, and anti-convulsant effects. Realgar mainly contains arsenic sulfide, which is antibacterial and resistant to blood-sucking worms. The main component of arsenic is arsenic trioxide, which can be used to treat leukemia and inhibit tumor angiogenesis [6].

Mercury is a toxic metal commonly found in mineral medicines. Inorganic mercury, elemental mercury, and methylmercury have strong binding to human serum proteins, and interfere with cell metabolism by binding to sulfhydryl groups of receptor proteins or cellular enzymes, thereby inducing cell death. Mercury can accumulate in vital organs such as the liver, kidneys, brain and heart with blood circulation. Arsenic also has significant cytotoxicity, and arsenic oxide can specifically damage pyruvate dehydrogenase in mitochondria, hindering both aerobic and anaerobic respiration. The cells thus suffer from acidosis and undergo rapid apoptosis. Long-term consumption of large amounts of realgar can produce obvious toxic reactions. One of the mechanisms responsible for the nephrotoxicity of realgar may be the accumulation of renal copper. However, there are also clinical methods for treating malignant diseases by inducing rapid apoptosis of cells.

Since the safety of toxic mineral drugs has always been controversial, there have been many cases of oral or topical poisoning of mineral drugs at home and abroad. Therefore, the Pharmacopoeia of the People's Republic of China has strict regulations on the use of toxic mineral drugs. For example, the incidence of adverse reactions caused by cinnabar increases with the increase of the dosage and the prolongation of the medication time. Therefore, cinnabar should not be taken in large quantities or a small amount for a long time, 0.1–0.5 g per day, mostly in pills and powder, not in decoctions, and appropriate for external use. The light powder should not be used in excess. It should be used with caution for internal use and appropriate amount for external use. In addition, it is also necessary to formulate corresponding drug restrictions for specific groups, such as pregnant women, children, etc., should pay attention to the contraindications and limits when using toxic mineral drugs.

The minerals of most Chinese medicines contain a mixture of many heavy metal elements. Most metal mineral drugs are hard in texture and difficult to be crushed into medicines. Therefore, they are usually processed by methods such as high-temperature calcination, the addition of auxiliary materials, grinding, water jetting, and purification before clinical application. The processing steps can also reduce the side effects of toxic mineral drugs. The composition and content of mineral medicines processed from the same mineral raw materials are different, which will lead to changes in their pharmacological effects. For example, the main component of gypsum is CaSO_4_·2H_2_O, which has a strong heat-clearing and clearing ability, while forged gypsum mainly contains calcium sulfate, which has a stronger hemostatic effect. The iron-containing mineral drug substitute ocher is calcined and quenched with vinegar to make the texture crisp and easy to crush. Among them, the content of toxic metals such as arsenic, lead, and mercury is significantly reduced, while the dissolution of iron, calcium, magnesium and other elements is significantly increased. The acute toxicity of realgar processed with yogurt was significantly reduced, but its analgesic and anti-inflammatory effects were the same as those of the raw product [7–9].

### Discussion on traditional preparations of mineral drugs

The medicament forms of TCM are an extremely characteristic part of the Chinese medicine culture. Meanwhile, it is also the product of TCM syndrome differentiation theory. The dosage forms of drugs always change with the nature of ailments and the physicochemical properties of the mineral drugs themselves. The great majority of mineral drugs are heavy and venomous. In traditional applications, most of them are prepared into pills, soups, and powders for the treatment of some difficult and miscellaneous diseases. Starting from the traditional dosage forms of TCM, this section discusses the application of mineral drugs in traditional medicine prescriptions and patent medicines. Table 2 shows detailed information on traditional mineral preparations.

**Table 2:** A summary of traditional preparations of mineral drugs

Preparation	Chinese patent medicine	Mineral medicines	Pharmacological action	Clinical application
Decoction	Baihu decoction	Gypsum	Clearing heat and promoting fluid production	Viral cold high fever, acute cerebral hemorrhage, infectious diseases, etc
	Zhengan Xifeng decoction	Os Draconis	Suppressing hyperactive liver for calming endogenous wind and nourishing yin for suppressing hyperactive yang	Hypertension, cerebrovascular disease, chronic gastritis, intractable insomnia, etc
	Dachengqi decoction	Mirabilite	Completed under the hot junction	Simple intestinal obstruction, adhesive intestinal obstruction, acute cholecystitis, acute pancreatitis, etc
	Bixie Shenshi decoction	Talc	Clearing heat and cooling blood and promoting diuresis	Gout, mycotic vaginitis, acute eczema, erythema nodosum, lower limb erysipelas
	Cream soup	Os Draconis	Relieving diarrhea with astringents	Chyluria, etc
	Chishizhi Yuyuliang decoction	Halloysite, Limonite	Astringing the intestine and arresting proptosis	Diarrhea, functional uterine bleeding, chronic enteritis, metrorrhagia, etc
	Guizhi Gancao Longgu Muli decoction	Os Draconis	Anti-depression, amelioration of menopausal syndrome	Insomnia, spontaneous sweating, night sweats, etc
	Jianling decoction	Ochre, Os Draconis	Nourishing yin for tranquillization and suppressing hyperactive liver for calming endogenous wind	Hypertension, cardiac neurosis hemorrhagic sinusitis, migraine, intractable hiccup, etc
	Ephedra Almond Licorice Plaster decoction	Gypsum	Antiviral, antipyretic, antitussive, expectorant, diuretic, sedative et al	Community-acquired pneumonia, pediatric acute bronchopneumonia, etc
	Zhuling decoction	Talc	Diuretic, antibacterial, improve renal function, inhibit the formation of kidney stones	Recurrent urinary tract infection, ascites due to hepatitis B cirrhosis, brain injury, etc
	Daqinjiao decoction	Gypsum	Anti-inflammatory	Gout, rheumatoid arthritis, facial palsy, scapulohumeral periarthritis, etc
	Xuanbai Chengqi decoction	Gypsum	Ventilating lung qi for dissipating phlegm and purgation	Pulmonary heart disease, asthma, bronchiectasis, lung abscess, etc
	Dahuang Mudan decoction	Mirabilite	Clearing heat for detumescence	Acute simple appendicitis, pelvic inflammation
	Xuanfu Daizhe decoction	Ochre	Harmonizing stomach for descending adverse qi	Gastritis, gastric distension, pyloric insufficiency, nervous hiccup, etc
	Huanglong decoction	Mirabilite	Clearing intestinal heat, replenishing qi, and nourishing blood	Typhoid fever, epidemic cerebrospinal meningitis, senile intestinal obstruction, etc
Pill	Jiebiao Zhuifeng pills	Cinnabar, Realgar	Dispel wind and dispel exterior, strengthen stomach and balance	Headache with wind-cold and cough
	Fangfeng Tongsheng pills	Mirabilite, Talc, Gypsum	Relieving superficies and catharsis and clearing heat and removing toxicity	Cold, trigeminal neuralgia, acute shigellosis, acute conjunctivitis, etc
	Niuhuang Qingwei pills	Gypsum	Improve gastrointestinal function and has a certain analgesic effect	Tonsillitis, gingivitis, recurrent oral ulcers, acute pharyngitis
	Niuhuangzhibao pills	Gypsum, Mirabilite, Realgar	Clearing heat-fire and facitating feces excretion	Functional constipation
	Liushen pills	Realgar	Antibacterial, anti-inflammatory, anti-viral, anti-convulsive, sedative, and enhance immunity	Chronic pharyngitis, chronic hepatitis, upper gastrointestinal cancer, leukemia, etc
	Jiuzhi Qingxin pills	Cinnabar, Realgar	Clearing heat-fire and facitating feces excretion	Dizziness, mouth and nose sores, sore throat, constipation knots, etc
	Liangge pills	Gypsum, Mirabilite	Anti-inflammatory and antipyretic	Sore mouth and tongue, hematemesis, cough, sore throat, etc
	Qingwei Huangliang pills	Gypsum	Antipyretic, anti-inflammatory, anti-viral	Stomatitis, periodontitis, and oral ulcer
	Huanglian Shangqing pills	Gypsum	Antipyretic, anti-inflammatory, antiviral, analgesic	Acute stomatitis, acute conjunctivitis, acute gastroenteritis, toothache mouth ulcer, etc
	Fufang Niuhuang Qingwei pillls	Gypsum, Mirabilite	Antipyretic, anti-inflammatory, and anti-viral	Acute pharyngitis, tonsillitis, oral ulcer, and periodontitis
	Daochi pills	Talc	Antibacterial, anti-inflammatory, analgesic, diuretic, antipyretic, anti-platelet aggregation, hemostasis	Stomatitis, urethritis, acute and chronic pyelonephritis, urinary calculi
	Jinsuo Gujing pills	Os Draconis	Decrease urine protein, regulate blood lipid and improve pathological changes of renal tissue	Male neurosis effects, chronic cervicitis, intractable night sweats, etc
	Wan`s Niuhuang Qingxin pills	Cinnabar	Clearing away the heat-evil and calming down	Japanese encephalitis, pertussis complicated with meningoencephalitis, etc
	Niuhuang Qingxin pills	Cinnabar, Realgar	Sedation, anti-convulsion, antipyretic, antihypertensive, improve hypoxia tolerance	Severe amnesia, aphthous stomatitis, intractable hiccups
	Angong Niuhuang pills	Cinnabar, Realgar	A calm, strong heart, protect brain tissue, antipyretic, anti-inflammatory, anti-pathogenic microorganisms	Japanese encephalitis, toxic pneumonia, high fever and coma caused by infection or poisoning, etc
	Suhexiang pills	Cinnabar	Dilate coronary artery, increase coronary flow, slow heart rate, reduce myocardial oxygen consumption, etc	Coronary heart disease angina, myocardial infarction, acute cerebrovascular disease, epilepsy, etc
	Mengshi Guntan pills	Mica-schist	Dispelling phlegm, relieving asthma and sedation, etc	Stroke, schizophrenia, epilepsy, migraines, neurosis effects
	Medical pills	Cinnabar, Realgar, Alum	Sedation, analgesia, anticonvulsants, etc	Epilepsy, manic psychosis
	Baizi Yangxin pills	Cinnabar	Inhibit the central nervous and play sedation, hypnosis, diazepam, anti-convulsion	Neurasthenia, memory loss, schizophrenia, menopause, hyperthyroidism, heart disease, etc
	Cizhu pills	Magnetite, Cinnabar	Nourishing blood for improving eyesight and tranquillization	For epilepsy, tinnitus, insomnia, and so on
	Tianwang Buxin pills	Cinnabar	Anti-myocardial infarction, enhance the body’s immune function, sedation, anti-convulsion, anti-arrhythmia, etc	Low blood pressure, insomnia, psychosis, hyperthyroidism, climacteric syndrome, chronic conjunctivitis, myocarditis, tuberculosis, hypertension, etc
	Zhusha Anshen pills	Cinnabar	Sedative-hypnotic, antiarrhythmic, anticonvulsant, antipyretic and analgesic, etc	Neurasthenia, schizophrenia, epilepsy, myocarditis, etc
	Naoliqing pills	Magnetite, Ochre	Lower blood pressure, sedation, antiemetic, vasodilator, etc	High blood pressure, arteriosclerosis, etc
	Zaizao pills	Cinnabar	Anti-coagulation, improve microcirculation, anti-inflammation, etc	Stroke, hemiplegia, numbness of hands and feet, pain and convulsion, etc
	Gejie Dingchuan pills	Gypsum	Antitussive, expectorant, anti-asthmatic, anti-inflammatory, antibacterial, enhance immune function	Bronchitis, asthma, tuberculosis, etc
	Suoyang Gujing pills	Os Draconis, Halite	Treatment of kidney deficiency	Male sexual neurosis effects, prostatitis, infertility, etc
	Shixiang Fansheng pills	Cinnabar, Mica-schist	Dissipating phlegm for resuscitation and tranquillization	Hysteria, neurosis effects, shock, epilepsy, schizophrenia, depression, seizures, etc
Powder	Liuyi powder	Talc	Clear away heat and facilitate urination	Heat and dampness caused by fever, external treatment of prickly heat, etc
	Qili powder	Cinnabar	Remove stasis and detumescence, relieve pain and stop bleeding	Soft tissue injury, and other infectious, purulent diseases, etc
	Zixue powder	Gypsum, Talc, Magnetite, Cinnabar, Mirabilite, North Calcium	Antipyretic, anticonvulsant, sedative, antibacterial, anti-inflammatory	Japanese encephalitis, sepsis, acute zinc phosphide poisoning, etc
	Biwen powder	Cinnabar	Get rid of the heat and the pain	Summer heat stroke, motion sickness, seasickness
	Bingpeng powder	Cinnabar, Borax, Sodium sulfate powder	Anti-inflammatory, anti-fungal, analgesic, etc	Gingival swelling and pain, tonsillitis, oral ulcers, mumps, herpes zoster, fungal vaginitis, and cervical erosion

### Traditional decoction of mineral medicine

The most scripture and oldest form of TCM is decoction. It has the characteristics of high-speed absorption and swift function. Mineral drugs are usually decocted with the pattern of pre-frying to fully stimulate the active ingredients. The reason is that most of the minerals are hard to dissolve or contain certain toxicity. Moreover, since the longtime of decocting, it can greatly reduce the toxicity. Baihu decoction (BHD) has the effect of clearing away heat and promoting body fluid with the gypsum as its sovereign drug. Modern research demonstrates that the material foundation of BHD is calcium ion in gypsum, in which 83.25% Ca exists in the free state and 23.79% Ca retains in soluble filtrate in the organic form [20]. By reducing the levels of interleukin-1β (IL-1β) and tumor necrosis factor α (TNF-α) in serum and hypothalamus of rabbits which was injected lipopolysaccharide, the BHD has an obvious antipyretic effect [21]. Therefore, BHD can become a natural anti-fever and anti-inflammatory candidate. BHD is diffusely used in the clinic to heal infectious diseases, viral poor and high fever, acute cerebral hemorrhage, diabetes, acute hyperglycemia, and rheumatoid arthritis [22, 23]. As shown in Table 2, Dachengqi decoction (DCQD) is a classical Chinese medicine recipe for curing constipation with excess heat. Mirabilite is one of the significant minister medicines, with positive heat-clearing and catharsis effects [24]. DCQD is constantly used in clinical therapy of acute cholecystitis, acute appendicitis, acute simple intestinal obstruction, and so on. It is reported that DCQD contributed with conventional therapy has a significant effect in the curing of hyperlipidemia acute pancreatitis [25]. Network pharmacology research found that DCQD on the therapeutics of pancreatitis may be achieved via two-way regulation, including anti-inflammatory and antioxidant effects, pro-apoptosis, and regulation of pancreatic secretion. This process involves numerous pathways and signals, such as interleukin-17 (IL-17), tumor necrosis factor (TNF), and nuclear factor kappa B, but the concrete mechanism of DCQD needs further study [26–28]. In clinical application, DCQD also illustrates the superiority in treating apoplexy, cerebral hemorrhage, and other diseases. From 296 active components of DCQD, 52 linchpin elements related to stroke were selected, which are mainly involved in the regulation of oxidative stress, lipid metabolism, and anti-inflammation [29]. Zeng et al. treated intracerebral hemorrhage model rats with DCQD for 7 days and found that the number of M2 microglia and N-methyl-D-aspartate receptors in the DCQD group increased significantly through the behavior test and protein subcomponent analysis. In this regard, the authors suggest that the mechanism may be through inhibiting mitogen-activated protein kinase and activating M2 microglia to protect rats with cerebral hemorrhage [30].

### Prescription preparation of mineral medicine

Based on the boundedness of clinical application of decoction, pills play an important role in mineral drug preparations. The reason is the easy storage, long efficacy, and convenient use of pills. The great majority of pills are designed for the therapeutics of some chronic and debilitating diseases, such as Zhusha Anshen pills containing HgS, Cizhu pills containing Fe_3_O_4_, etc. And they all possess the characteristics of slow absorption, lasting curative effect, for epilepsy, insomnia, tinnitus has a superior therapeutic effect. Some of the pills prepared from certain aromatic or toxic minerals can also make it in fast treatment, such as Angong Niuhuang pills and Suhexiang pills. Suhexiang pills, which are mainly composed of cinnabar, have a positive protective effect on the acute ischemic lesion of myocardial cells. It has been found that the Suhexiang pill can dilate coronary arteries and reduce myocardial oxygen consumption [31]. Spontaneously, it is commonly used in the clinical treatment of coronary heart disease angina pectoris, myocardial infarction, and other emergencies. With the discovery of a novel drug matrix, pills have shown great potential in sustained-release and controlled-release preparations. Rational platform design can maximize the efficacy of mineral drugs and minimize their adverse effects.

The powder is divided into external and internal use of two, mineral medicine oral powder is generally developed into a certain size of dust and taken with water or decoction. The powder has the virtues of both decoction and pills, which have high-speed absorption and rapid activity. For some powerful and mostly toxic mineral drugs, the oral powder is generally used in some emergency treatment, and there are strict dosage restrictions. For example, the dosage of HgS cannot exceed 0.5 g, and As_2_S_2_ cannot outstrip 0.1 g. Zixue, a powder with excellent clinical curative effect, contains a large number of minerals, including gypsum, north calcitum, talc, magnetite, mirabilite, cinnabar, etc. Modern research shows that it has antipyretic, anticonvulsant, sedative, antibacterial, anti-inflammatory, and other effects, and represents excellent results in the treatment of Japanese encephalitis, epidemic cerebrospinal meningitis, sepsis, and other acute diseases. Qili powder is another typical mineral powder preparation, and cinnabar is one of the eventful components. Qili powder has the functions of removing blood stasis, detumescence, relieving pain, and hemostasis. It is commonly used in soft tissue injury, fracture, and other diseases. Consult with the traditional dosage forms of mineral drugs can provide research direction and ideas for the modern development of mineral drugs.

### Research on mineral drug delivery systems

**Fig. 3:**
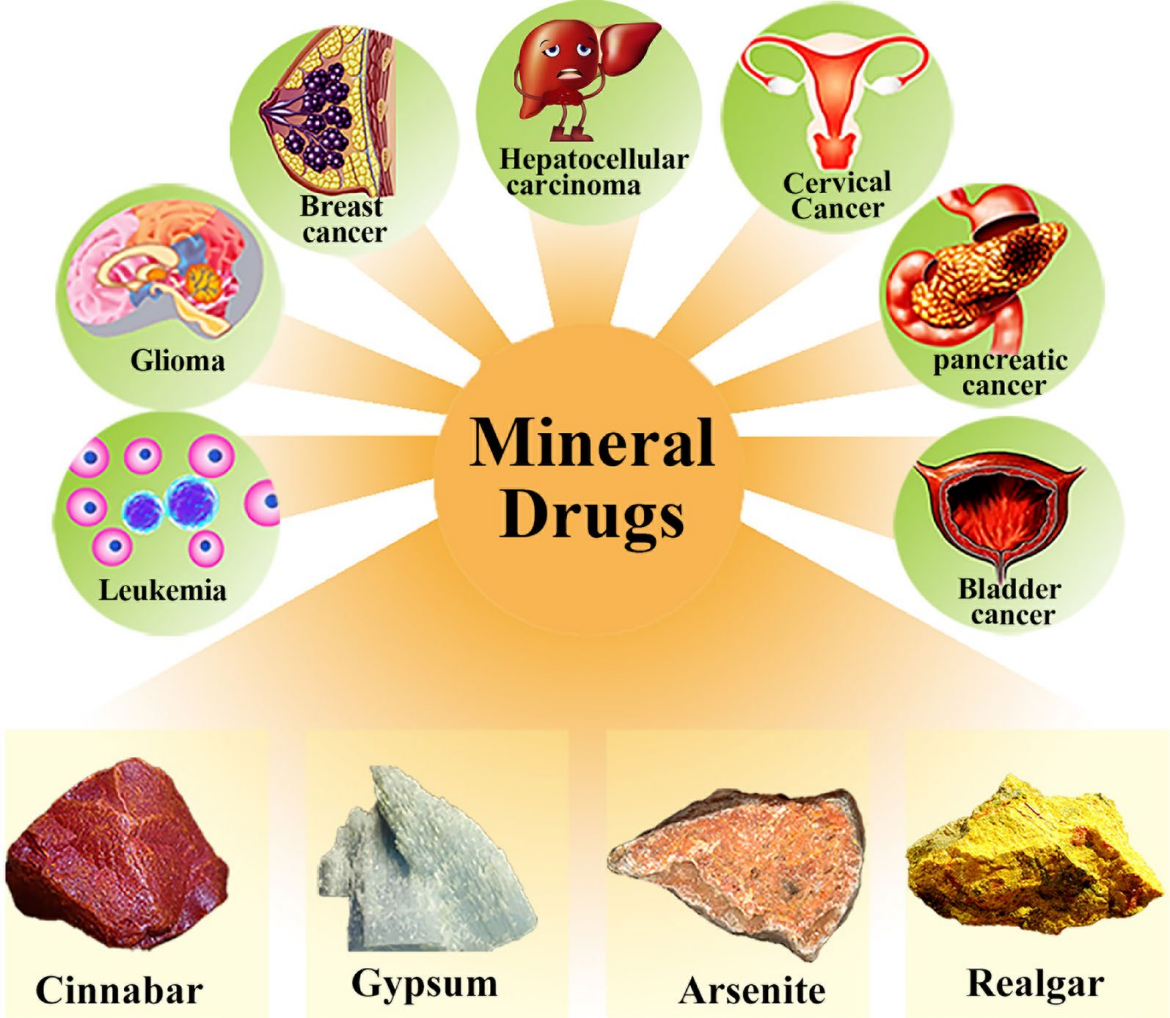
Schematic illustration showing the pharmacological activity of mineral drugs and their advantages in tumor treatment. (The copyright of the pictures from network sources in the figure is shown below. Copyright^©^ 2021 Sohu All Rights Reserved. Copyright^©^1997–2021 https://www.163.com/.)

The development of nano-drug delivery systems conducts opportunities for the growth and research of TCM, especially the application of mineral drugs. And it cannot be ignored, and always has the significance of TCM in the diagnosis and treatment of diseases. Exploration of active components and pharmacological effects of mineral drugs has always been the focus of scholars ' research. Accordingly, there are a few studies on the delivery system of mineral drugs. Modern pharmacological studies have shown that mineral drugs such as arsenic trioxide and realgar have an active therapeutic effect on a variety of malignancies, including acute promyelocytic leukemia (APL), liver cancer, gastric cancer, and cervical carcinoma (Fig. 3), as shown in Table 3. Therefore, the research of mineral drugs in modern drug delivery systems is discussed in detail in this section. Firstly, the research of mineral drug delivery in nano-drug delivery was discussed from the aspects of APL, hepatocellular carcinoma (HCC), and other diseases. Then, taking magnetite as an example, the application of mineral drugs as carriers in the study of drug delivery systems is discussed.

**Table 3:** Novel preparations of mineral drugs and preclinical study

Register number	Study title	Conditions	Interventions/drugs	Locations
NCT04687176	Frontline oral arsenic trioxide for APL	APL	Dru: oral arsenic trioxide formulation	The University of Hong Kong
NCT03624270	Oral arsenic trioxide for newly diagnosed acute promyelocytic	APL	Drug: oral arsenic trioxide, all-trans retinoic acid, and ascorbic acid	The University of Hong Kong
NCT04689815	Oral arsenic trioxide for nucleophosmin 1 mutated acute myeloid leukemia (AML)	AML	Drug: oral arsenic trioxide formulation	The University of Hong Kong
NCT04634838	Efficacy dressings with copper oxide	Pressure ulcer	Device: medcu antibacterial wound dressings with copper oxide	Loewenstein Rehabilitation Center ra'anana, Israel
NCT01565798	Efficacy of copper to reduce acquisition of microbes and healthcare-acquired infections	Healthcare-acquired infection	Other: copper-alloy surfaced patient care objects	Medical University of South Carolina
NCT01678612	Efficacy of copper in reducing health-acquired infections in a pediatric intensive care unit	Nosocomial infections	Copper-alloy surfaced objects	Hospital Roberto del Rio, Santiago, Chile
NCT03323346	Phase II trial of disulfiram with copper in metastatic breast cancer	Breast neoplasm female	Drug: disulfiram	University hospital Olomouc, Olomouc, Czechia
NCT03284749	Effect of copper on the healing of obstetric wounds	Cesarean section and infection	Other: copper impregnated wound dressing, and normal wound dressing	Croydon health services NHS trust
NCT04511468	Zinc, chromium, vitamin C, and copper combination supplement for prediabetes progression	Prediabetes	Dietary supplement: zinc, chromium, vitamin C, and copper supplementation, and placebo	Human Nutrition Research Center
NCT01777919	Disulfiram/copper combination in the treatment of newly diagnosed glioblastoma multiform	Glioblastoma multiforme	Temozolomide, disulfiram, copper	Olympion medical Center, Patras, Greece
NCT00608946	Treatment with copper in patients with mild Alzheimer’s dementia	Alzheimer’s disease	Dietary supplement: copper	
NCT03736109	Role of a copper-albumin complex in the treatment of knee osteoarthritis in human	Osteo arthritis knee	Genetic: soluble and genetic biomarkers measurements	
NCT02715609	Disulfiram/copper with concurrent radiation therapy and temozolomide in patients with newly diagnosed glioblastoma	Glioblastoma multiforme	Drug: disulfiram, and copper gluconate	Washington University school of medicine, Saint Louis, Missouri, United States
NCT01017315	Efficacy of baby talcum in the prevention of pruritus assosiated with cast	Fracture distal radius	Drug: baby talcum	Prince of Songkhla university
NCT04792970	Randomized controlled trial of talc instillation in addition to daily drainage through a tunneled pleural catheter to improve rates of outpatient pleurodesis in patients with malignant pleural	Malignant pleural effusion	Drug: talc	Duke University Medical center, Durham, North Carolina, United States
NCT00789087	Talc pleurodesis in patients with recurrent malignant pleural effusion	Recurrent malignant pleural effusion	Procedure: video thoracoscopic talc poudrage, and talc slurry through a chest tube	University of são Paulo medical school
NCT03635138	Effect of the incoportation of copper and zinc nanoparticles into dental adhesives	Caries, dental	Other: metal nanoparticles (Zn and Cu), and dental adhesive pure	Eduardo Fernández G, Santiago, Metropolitana
NCT04775238	Effect of metallic nanoparticles on nosocomial bacteria	Nosocomial infections	Other: silver nanoparticles, and copper nanoparticles	Faculty of medicine-sohag University, Sohag, Egypt
NCT02200978	A study for improving the outcome of childhood acute promyeloid leukemia	Childhood acute promyelocytic leukemia	Drug: indigo naturalis formula, and ATO, ATRA (and 5 more…)	The first affiliated hospital of Sun Yat-Sen University
NCT04489706	Arsenic trioxide in recurrent and metastatic ovarian cancer and endometrial cancer with P53 mutation	Ovarian cancer	Drug: arsenic trioxide for injection	Shanghai Jiaotong university school of medicine affliated ruijin hospital
NCT02966301	Treatment of chronic graft versus host disease with arsenic trioxide	Chronic graft-versus-host disease	Drug: arsenic trioxide injectable solution	CHU de caen-institut d'Hématologie de basse normandie
NCT01470248	Study of arsenic trioxide in small cell lung cancer	Lung cancer	Drug: arsenic trioxide	Emory University Winship Cancer institute
NCT01791894	Arsenic trioxide in treating patients with basal cell carcinoma	Basal cell carcinoma of the skin	Drug: arsenic trioxide	Stanford university medical center
NCT03751917	Long-term safety study of arsenic trioxide in newly diagnosed, low-to-intermediate risk acute promyelocytic leukemia	Acute promyelocytic leukemia	Drug: Arsenic Trioxide	Aon Ss. Antonio E Biagio E C. Arrigo-Alessandria-soc ematologia,Alessandria, Italy
NCT00009867	Arsenic trioxide in treating patients with urothelial cancer	Recurrent transitional cell cancer of the renal pelvis and ureter	Drug: arsenic trioxide	Cancer and leukemia group B
NCT00024258	Arsenic trioxide in treating patients with advanced neuroblastoma or other childhood solid tumors	Brain and central nervous system tumors	Drug: arsenic trioxide	Memorial Sloan-Kettering Cancer Center, New York
NCT00008697	Arsenic trioxide in treating patients with refractory or recurrent acute promyelocytic leukemia	Leukemia	Drug: arsenic trioxide	Washington university school of medicine
NCT00045565	Arsenic trioxide plus radiation therapy in treating patients with newly diagnosed malignant glioma	Adult giant cell glioblastoma	Drug: arsenic trioxide	New approaches to brain tumor therapy consortium, Baltimore
NCT00517712	Single-agent arsenic trioxide in the treatment of newly diagnosed acute promyelocytic leukemia	Acute promyelocytic leukemia	Drug: single-agent arsenic trioxide	Kidwai memorial institute of oncology, India
NCT00053222	Arsenic trioxide in treating patients with pancreatic cancer that has not responded to gemcitabine	Pancreatic cancer	Drug: arsenic trioxide	Decatur memorial hospital cancer care institute, Decatur, Illinois, United States
NCT00193518	Arsenic trioxide in relapsed/refractory chronic lymphocytic leukemia/small lymphocytic lymphoma	Chronic lymphocytic leukemia	Drug: arsenic trioxide	Tennessee Oncology, Nashville, Tennessee, United States
NCT00128596	Arsenic trioxide in treating patients with metastatic liver cancer that cannot be removed by surgery	Liver cancer	Drug: arsenic trioxide	UPMC cancer centers, Pittsburgh, Pennsylvania, United States
NCT00003630	Arsenic trioxide in treating patients with advanced solid tumors	Unspecified adult solid tumor, protocol-specific	Drug: arsenic trioxide	Memorial Sloan-Kettering cancer center, New York
NCT00075426	Arsenic trioxide in treating patients with locally advanced or metastatic non-small cell lung cancer	Lung cancer	Drug: arsenic trioxide	University of texas medical branch
NCT00005069	Arsenic trioxide in treating patients with metastatic kidney cancer	Kidney cancer	Drug: arsenic trioxide	Memorial Sloan-Kettering cancer center New York
NCT00005040	Arsenic trioxide in treating patients with relapsed or refractory non-Hodgkin's lymphoma	Lymphoma	Drug: arsenic trioxide	Memorial Sloan-Kettering cancer center New York
NCT00036842	Arsenic trioxide in treating men with germ cell cancer	Extragonadal germ cell tumor testicular germ cell tumor	Drug: arsenic trioxide	MBCCOP-Gulf coast mobile, Alabama, United States
NCT03503864	Phase II study of combined chemotherapy with arsenic trioxide in stage 4/M neuroblastoma	Neuroblastoma	Drug: arsenic trioxide	Sun Yat-sen memorial hospital, Sun Yat-sen University
NCT04869475	Arsenic trioxide in refractory solid tumors with rescuable p53 mutation	Refractory solid tumors	Drug: arsenic trioxide	Department of Oncology, Ruijin hospital
NCT00621023	Cephalon decitabine, arsenic trioxide, and ascorbic acid for myelodysplastic syndrome	Myelodysplastic syndrome	Drug: decitabine, arsenic trioxide, and ascorbic acid	Duke university medical center

### Application of mineral drugs in tumor remedy

#### Acute promyelocytic leukemia

Acute promyelocytic leukemia is a common malignant disease of hematopoietic tissue. T (15, 17) chromosome rearrangements lead to this malignant tumor. APL is characterized by representative promyelocytic leukemia protein/retinoic acid receptor a gene fusion and high invasiveness and infiltration in clinical practice [32–35]. The oncogenic protein produced by the fusion can antagonize myeloid differentiation and promote APL to initiate cell self-renewal, leading to abnormal development of promyelocytic bone marrow infiltration [36, 37]. Owing to high early mortality, high recurrence rate, low survival rate, and coagulopathy. It was once considered to be the most rapidly fatal leukemia [32]. Although all-trans retinoic acid (ATRA) and anthraquinone chemotherapy are recognized as effective therapies for APL, their side effects such as cardiotoxicity cannot be ignored [38]. Therefore, scientists are always devoted to finding ways to validly remedy APL. In the past 60 years, significant progress has been made in the treatment of APL. The introduction of ATO holds promise for the treatment of APL.

**Fig. 4:**
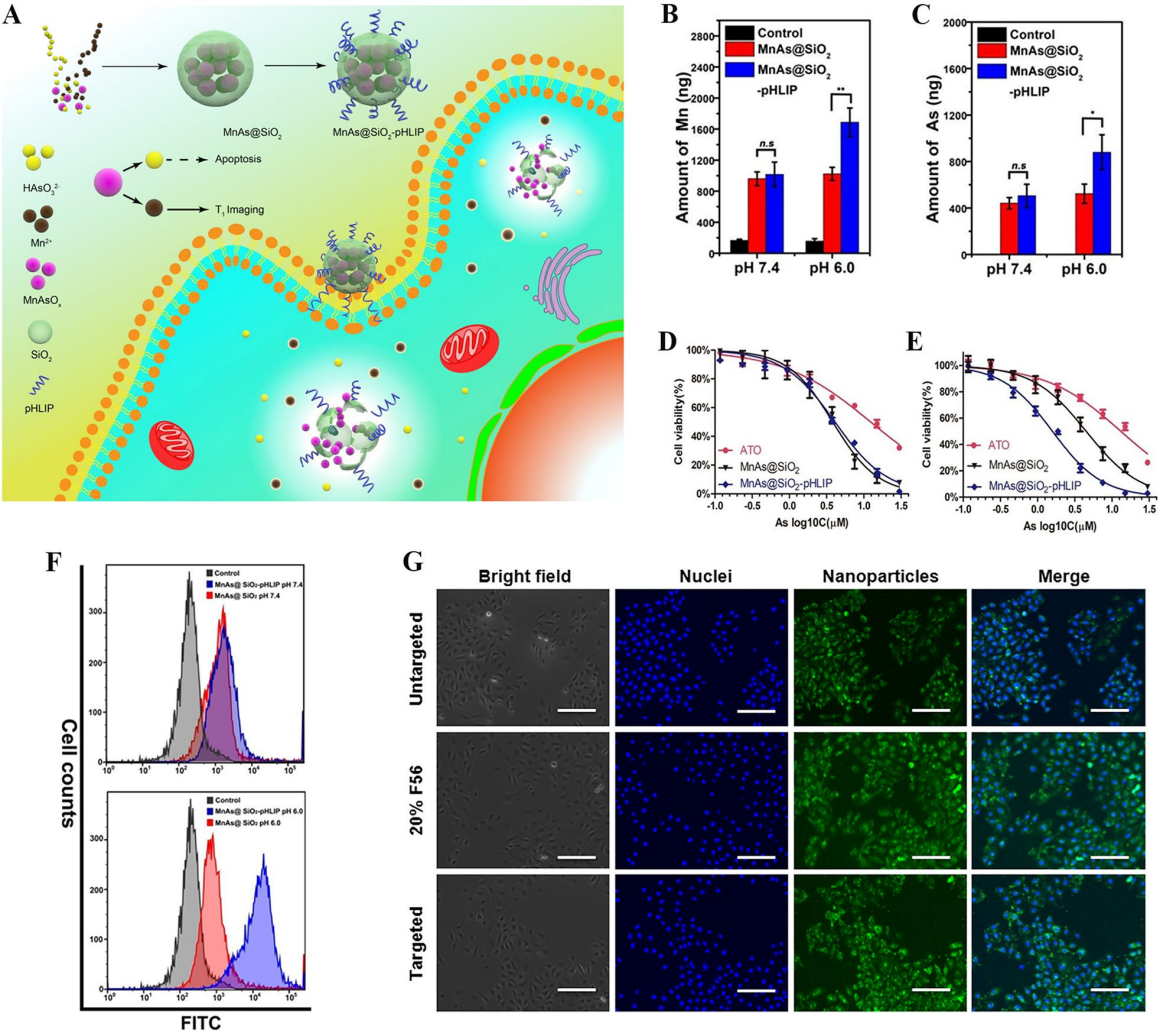
**A** Schematic illustration of the synthesis of MnAs@SiO2-pHLIPs and their functions in the tumor. After treating SMMC-7721 cancer cells with MnAs@SiO_2_ and MnAs@SiO_2_-pHLIP, the total amount of Mn ions (**B**) and As ions (**C**) in the cells at pH 7.4 and pH 6.0. **F** Flow cytometric analysis of SMMC-7721 cells incubated with different drugs for 2 h at different pH. SMMC-7721 cells served with free ATO, MnAs @ SiO_2_, and MnAs @ SiO_2_ pHLIP at pH 7.4. **D** And the cell viability at pH 6.0 (**E**) and pH 7.4. (*n* = 6) [40] Copyright 2019, Royal Society of Chemistry. **G** Fluorescence microscopy images of prepared oligopeptide nanoparticles in HUVEC cells (scale bar:200 μM). [41] Copyright 2020, Elsevier

Arsenic has always been regarded as the king of poisons. However, its active ingredient ATO has recently been considered as one of the most effective drugs for the treatment of APL. As early as the 19th and early twentieth centuries, Western doctors found that arsenic had special effects in the treatment of leukemia, and adopted a variety of treatment methods such as oral, rectal, intramuscular and intravenous administration. Arsenic trioxide has been widely used in the world since the 1990s to treat acute promyelocytic leukemia. It is an original invention of Chinese and Western medicine experts from Harbin Medical University in China. The use of hemoglobin can show extraordinary binding force with exogenous drugs, so that trivalent arsenic can be combined with hemoglobin. In addition, tumor cells will absorb nutrients preferentially to normal cells in the tissue, so that trivalent arsenic is mainly absorbed by tumor cells. destroy tumor cells. In addition, hemoglobin has the advantages of low cost, complete biocompatibility, and easy genetic engineering, all of which make hemoglobin an ideal drug delivery material. Fan et al. designed nanoparticles composed of polyethylene glycol (PEG), retinoic acid (RA), and 2-chloro-1,3,2-dioxaarsolane (PEG-As-RA), which exhibited superior water solubility, stability, and biocompatibility [39]. Zhang et al. developed an ATO multifunctional drug delivery system based on a pH-low insertion peptide modification, which is termed as MnAs@SiO_2_-pHLIP (Fig. 4A). Figure 4B, C, F illustrate that arsenical and manganese ions can aggregate efficiently in cancer cells. This drug delivery system can controllably release loaded ATO in response to an acidic environment, thereby promoting the apoptosis of cancer cells in Fig. 4D, E [40]. The application of protein peptides provides an efficient platform for the delivery of ATO. Fan et al. showed that double oligopeptide nanoparticles based on the principle of reverse micelles can significantly increase the loading rate and stability of ATO (Fig. 4G). This bone marrow targeted drug delivery strategy may be utilized to treat chronic myeloid leukemia and other hematological malignancies originating from bone marrow [41]. Zn-based metal–organic framework zeolitic imidazolate framework-8 has proved to be a suitable candidate material for transporting arsenic compounds, which could not only enhance the transportation of ATO but also possess pH-responsive release capability [1]. Otherwise, Jin et al. loaded arsenite into liposomes (LIP) by electrostatic interaction with manganese ion to construct liposome-encapsulated arsenic manganese complex (LIP@MnAs_x_). The research results show that LIP@MnAs_x_ is a desired arsenic-based nano-drug system, which can improve the non-specific distribution of NaAsO_2_ in the body [2]. These all provide us with ideas for delivering ATO to treat APL.

#### Glioma

Glioma is the most common primary intracranial disease with invasiveness growth, poor prognosis, high recurrence rate, and high mortality [42]. Multi-mode therapy such as chemotherapy, radiotherapy, and surgical resection are the main choices for the treatment of gliomas [43, 44]. However, the emergence of drug resistance and the presence of blood–brain barriers (BBB) continue to be problems impeding the mission of drugs. At the same time, the application of drug chemotherapy often generates irreversible damage to the body, and patients are usually accompanied by severe adverse reactions and pain. In addition, after brain surgery to remove tumor tissue, the residual tissue still has a trend of growth and invasion, resulting in a high recurrence rate [45]. Therefore, it is crucial to exploit new drugs and modus for the treatment of gliomas.

**Fig. 5:**
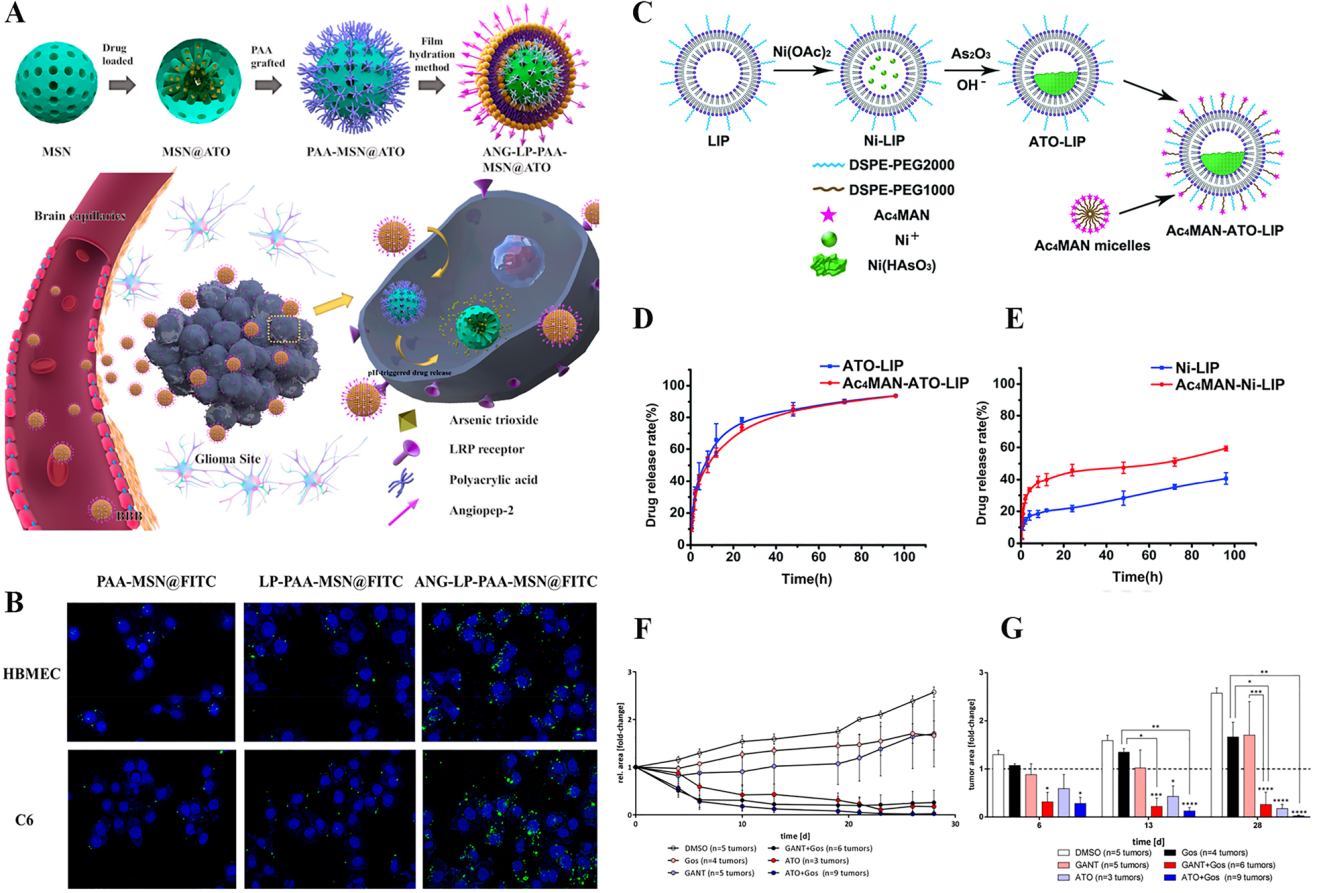
**A** Schematic illustration for the synthesis and preparation of ANG-LP-PAA-MSN@ATO for glioma therapy. **B** Confocal images of HBMEC cells and C6 cells treated with the three agents for 4 h [48]. Copyright 2019, American Chemical Society. **C** Schematic represented for ATO liposome modified with Ac4MAN micelle. **D** Drug release curves of ATO-LIP and Ac4MAN-ATo-LIP (**E**) drug release curves of Ni-LIP and Ac4MAN-Ni-LIP (*n* = 3) [49]. Copyright 2019, American Chemical Society. **F** Tumor growth curves after treatment with the same dose of dimethyl sulfoxide, GANT, ATO, Gos, GANT/Gos, and ATO/Gos at 2.5 µM. **G** Bar charts made at 6, 13, and 28 days [50]. Copyright 2019, MDPI

It is well known that arsenic and its compounds are highly toxic, especially trivalent arsenic and pentavalent arsenic. Further studies have shown that ATO has an inhibitory effect on intractable diseases, including gliomas. The anti-cancer mechanisms of ATO include regulating apoptosis and autophagy, promoting the production of intracellular reactive oxygen species, and inhibiting tumor stem cells [46, 47]. Unfortunately, there are still many problems in ATO therapy, such as severe adverse reactions, high organ clearance, and poor blood–brain barrier permeability. Therefore, the design of drug carriers to overcome the BBB has become the focus of research. Tao et al. grafted polyacrylic acid (PAA) onto mesoporous silica nanoparticles (MSN) and combined them with angiopep-2-liposomes to prepare nanocarrier (ANG-LIP-PAA-MSN) (Fig. 5A) [48]. Figure 5B shows that modified agents are better absorbed by cells. Otherwise, Wang et al. showed that mannose (MAN)substrates without acetyl groups can promote the entry of vesicles into the brain, while the surface of LIP undergoes deacetylation of the Ac4MAN group under the action of enzymes, which is the “prodrug-like” characteristic of vesicles. Therefore, they insert distearoyl phospho-ethanolamine-polyethylene into the liposomes, thereby developing the "prodrug-like" ATO liposomes called Ac4MAN-ATO-LIP (Fig. 5C) [49]. Figure 5D, E show that liposomes modified with surfactants have a better drug release rate. To strongly suppress tumor growth or even tumor removal, Linder et al. designed a combination therapy consisting of the hedgehog signaling pathway inhibitors GANT61, anticancer drug gossypol (Gos), and ATO in Fig. 5F, G [50]. These provide a promising targeted therapy for the treatment of glioma.

In addition, modifying groups on drug carriers can enhance the targeting ability by recognition and combination of the BBB, including angiopep-2 (ANG) and TGN peptides [48, 51, 52]. With the help of these groups, drugs are commendably accumulated in the tumor tissue area and play a role in targeted therapy. Other tumor-targeted modified peptides also ulteriorly enhance the penetration of the preparation, such as the internalizing RGD (iRGD, CRGDK/RGPD/EC) peptides. IRGD peptides can bind to the neuropilin-1 receptor of tumor cells, thus reducing cellular internalization. This suggests that we can design dual targeting nanomedicines to the blood–brain barrier and tumor. And using different nano-drug platform strategies according to different diseases is the trend of nanomedicine development in the future.

#### Hepatocellular carcinoma

The most popular pernicious tumor in the world is must belong to hepatocellular carcinoma, which is ranked the third one in cancer death. In the past decades, the hepatitis B virus (HBV) has been confirmed to be the main pathogenic factor. About 50–80% of liver cancer cases in the world are caused by HBV infection [53, 54]. Studies have shown that HBV infection can directly or indirectly lead to HCC through coinfection with hepatitis C virus, heavy drinking, or type 2 diabetes. The main mechanisms include HBV gene amalgamation, genomic uncertainty induced by variant, and activation of tumor signal pathway [55]. As the result of intense malignant and occultation of hepatocellular carcinoma, many patients with liver cancer have unsatisfactory surgical results, and the recurrence rate and mortality are high. Furthermore, HCC has high drug resistance in conventional chemotherapy and radiotherapy [56]. Therefore, it is a great challenge to develop therapeutic methods that can improve the efficacy and decrease the toxicity of HCC. ATO is a mineral drug with a positive curative effect on a variety of tumors. It has the characteristics of multi-target and multi-efficacy of TCM and has been proved to have a positive effect on multifarious tumors. Nadra et al. studied the irradiation of normal liver and hepatoma cells (Hep3B) at 24,48, and 72 h with different consistencies of ATO [57]. And the results show that ATO is promising to be a predominant drug for the curing of HCC.

**Fig. 6:**
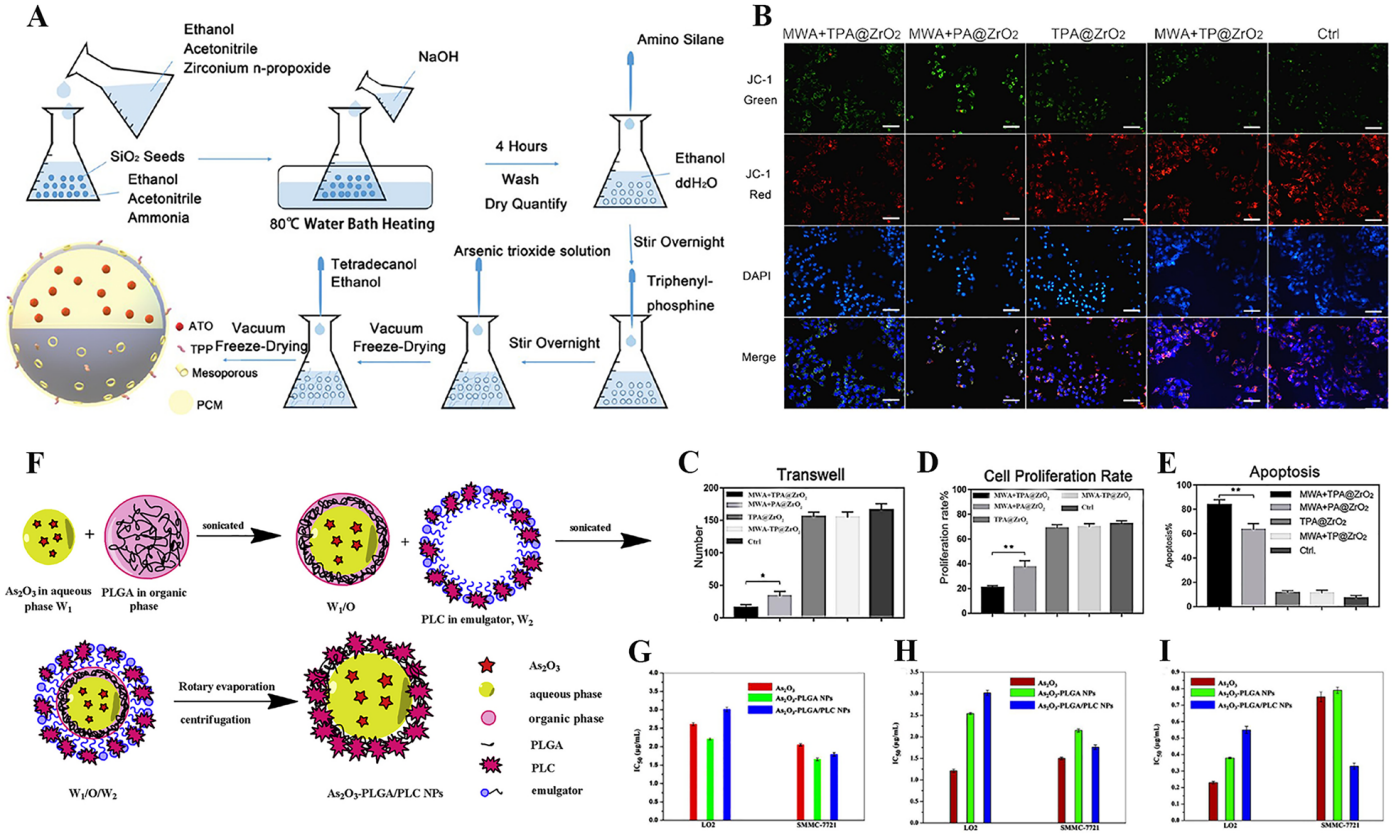
**A** Construction of the composite nanodrug delivery system. **B** JC-1 mitochondrial membrane potential staining (200×). Cell migration (**C**), proliferation (**D**), and apoptosis (**E**) test results after treatment with the composite nanoparticles [61]. Copyright 2020, American Chemical Society*.* **F** The synthesis scheme of As_2_O_3_-PLGA/PLC NPs. IC50 values of the nanoparticles against LO2 and SMMC-7721 cells after 24 (**G**), 48 (**H**), and 72 (**I**) h incubation (mean ± SD, *n* = 5) [3]. Copyright 2019, Elsevier

However, on account of the systemic toxicity, swift renal elimination, and greater serum protein binding rate of ATO [58], ATO is seriously limited in the treatment of HCC [59]. We know that reducing the size of nanoparticles and preparing smaller ATO nanoparticles can improve the ability of nanoparticles to penetrate BBB and cell membranes. ATO microcrystalline microspheres with very high-density cores were prepared by embedding ATO microcrystalline with polylactide glycolic acid. ATO micro-crystal microspheres can continuously induce the production of reactive oxygen species and apoptosis of liver carcinoma cells. And reduce tumor growth by 80% through local administration. It realizes local management of ATO, avoids systemic exposure, and possesses higher pharmacological stress [60]. In recent years, chemotherapy combined with phototherapy and hyperthermia has obvious advantages in tumor treatment. Wu et al. developed a mitochondria-targeted carrier with local precisely controlled release of ATO by loading ATO into the cavities of nano zirconia microspheres [61]. After the ATO nano-drug was delivered to the tumor site through the enhanced permeability and retention effect, the tetradecyl alcohol was used as a phase change material (PCM) in vitro to be exposed to triphenylphosphine (TPP) by microwave control in vitro phosphine targets the ligand to enhance the mitochondrial killing effect of ATO. A kind of mitochondrial-targeted ATO controlled release carrier (TPP-PCM-ATO@ZrO_2_) was constructed by coupling microwave-sensitive smart switches and mitochondrial targeting molecules outside the microspheres (Fig. 6A). The developed drug delivery system has (1) strong permeability and can inhibit tumor cell proliferation and promote apoptosis Fig. 6C–E, (2) controlled release of ATO in tumors, (3) ligands that can point mitochondria in Fig. 6B, (4) microwave therapy via an extracorporeal approach for improved efficacy. However, the mechanism of ATO mingled with microwave hyperthermia needs further study. Chitosan is a natural polysaccharide. Because of its good biocompatibility, biodegradability, and cytotoxicity to cancer cells, chitosan has become an ideal material for tumor treatment [62]. It is reported that by encapsulated ATO in PEG and lactic acid-modified chitosan (PLC). A nano-drug targeting ATO release from liver cancer cells was then prepared by coating with poly (lactide-*co*-glycolide) (PLGA) nanoparticles (As_2_O_3_-PLGA/PLC NPs) (Fig. 6F). The prepared nano-drugs have good targeting to hepatocellular carcinoma and can effectively release ATO to cancer cells. Experimental studies showed that As_2_O_3_ PLGA/PLC NPs had an obvious inhibitory influence on human hepatoma cell lines SMMC-7721, but had low cytotoxicity on normal human hepatocytes (LO2 cells) in Fig. 6G–I [3]. Therefore, PLGA/PLC nanoparticles are a safe and effective drug carrier for liver cancer chemotherapy. Recent research reported the development of magnetic targeted nano-carrier technology has brought a new research direction for the effective transportation and release of ATO. Approach by modifying ATO prodrugs (NiAsO_x_) loaded magnetic large-pore mesoporous silica nanoparticles (M-LPMSNs) with folic acid (FA), the responsiveness was prepared to released nano drugs (M-LPMSN-NiAsO_x_-FA). Further experiments show that the prepared nanomedicine has stronger cytotoxicity and the ability to promote cell apoptosis than the traditional free ATO [63]. ATO-containing magnetic silica nanoparticles can not only effectively resist hepatocellular carcinoma, but also have the ability to detect tumors in real-time. It has great application prospects and advantages in HCC treatment.

#### Cervical carcinoma

Cervical cancer is a common malignant tumor of the female reproductive system with a high incidence [64]. There are 500,000 new cases annually in the world. Comprehensive treatment including surgery is the main principle of treatment in the clinic. However, the cure rate of postoperative recurrence is less than 20%, in addition, chemotherapeutic drugs lack targeting and there are many side effects [65]. As there is no breakthrough in the comprehensive treatment of advanced cervical cancer, insights into the traditional mineral drug of ATO may be an alternative manner in cervical cancer treatment.

**Fig. 7:**
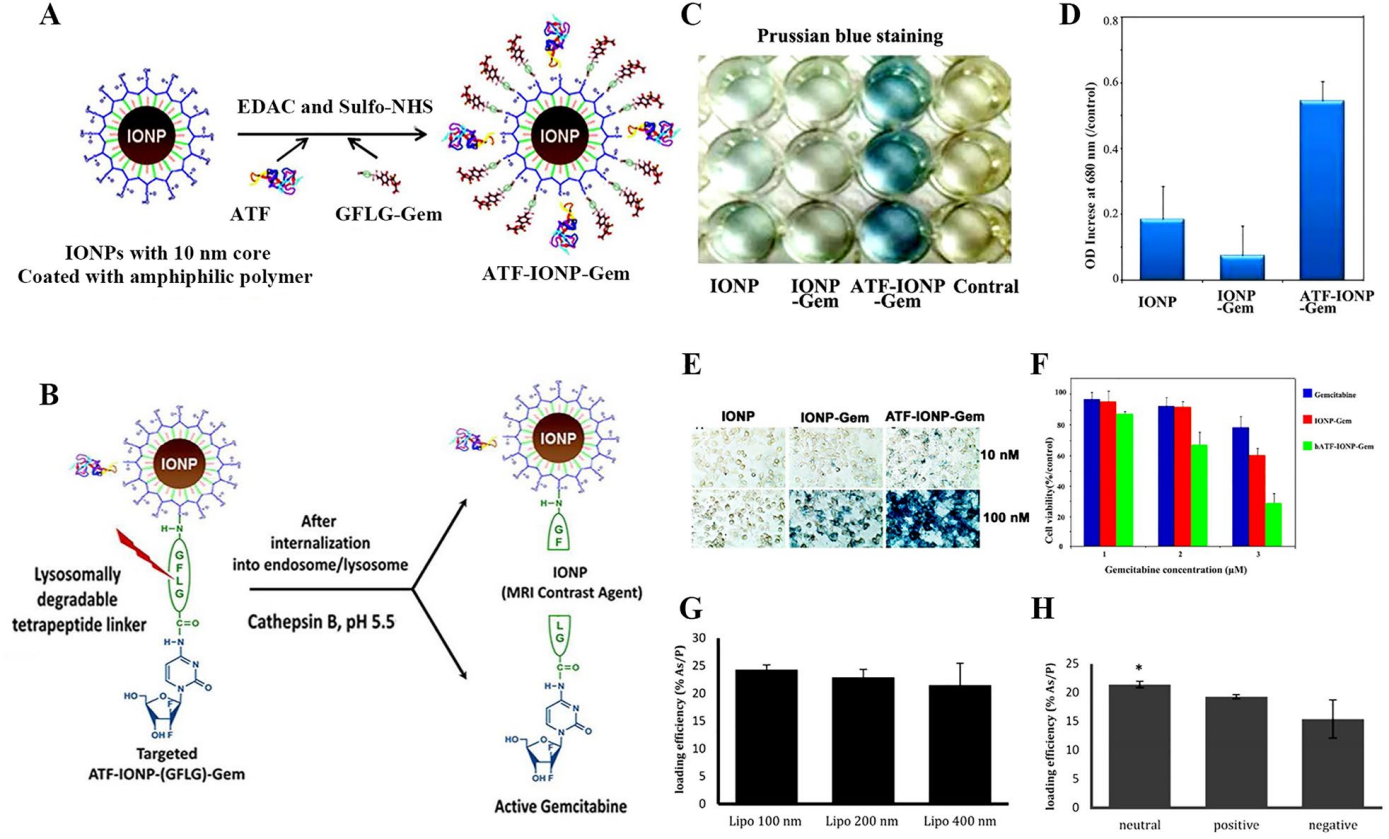
**A** Schematic of the preparation of ATF-IONP-Gem. **B** Drug release condition and efficiency. IONPs, IONP-Gem, ATF-IONP-Gem, and human pancreatic cancer cells were incubated in 100 nm and 10 nm for 4 h, then Prussian blue staining was performed. **C** IONPs in cells were detected as blue-stained cells. **D** the OD value was quantitatively detected at 680 nm with a micro board reader. **E** The observed bright-field microscopic images of Prussian blue-stained cells under 40 × magnifications of. **F** the number of living cells per well in a 96-well tissue culture plate was determined by the MTT method [76]. Copyright 2013, American Chemical Society. **G** The drug loading rate of synthetic liposomes was determined by inductively coupled plasma optical emission spectrometry (ICP-OES) at different particle sizes and (**H**) different charges [64]. Copyright 2019, MDPI

ATO has been proved to be effective in many cancers including cervical cancer. However, there is a lack of experiments to verify the anti-cervical cancer mechanism. Nowadays, Zhang et al. preliminary predict that ATO can induce reactive oxygen production to promote cell apoptosis by methyl thiazolyl tetrazolium (MTT) and flow cytometry. Western blotting and flow cytometry indicated that ATO suppresses hypoxia‑inducible factor‑1α expression, which aimed to effectively inhibit the proliferation and invasion of Siha cells and promote apoptosis [65]. ATO due to the dose-limited toxicity and poor pharmacokinetics in clinical trials requires the right vehicle to be carried [66]. The liposome is a common drug delivery carrier to encapsulate ATO in cervical cancer treatment. Liposomal ATO has been proved to exert better efficacy and lower cytotoxicity. At the same time, liposomes are regarded as a reservoir of drugs, and the slow release of drugs can avoid the toxic and side effects of high concentrations of ATO. Wang et al. showed that compared with free ATO, liposomal ATO further reduced the level of human papillomavirus-E6 protein and induced apoptosis of HeLa cells [67]. In addition, the concentration of arsenic in cells was measured by an inductively coupled plasma optical emission spectrometer (ICP-OES). Results showed that the intracellular concentration of ATO transported by liposomes was 3 times lower than that of free ATO, which verified the slow accumulation of ATO and lower cytotoxicity. The liposomes with different particle sizes and charges also have an impact on efficacy. Therefore, Akhtar utilized ICP-OES for the quantitative analysis of arsenic. Figure 7G, H show that neutral liposomes of 100 nm were the highest drug loading rate, which was 24.2%As/P (± 0.848) [64]. In addition, the cytotoxicity test showed that neutral liposomes were the least toxic.

#### Other nano-preparations

The development of biomimetic nanoparticles and targeted drug delivery technology has opened up a new research path for mineral drugs. And the components of traditional Chinese medicine and mineral drugs are gradually attracting attention in the drug delivery system. For example, antigen and antibody recognition has high specificity and affinity, which is a very ideal targeted drug delivery carrier. CD44v6 is a membrane antigen overexpressed in epithelial tumors such as pancreatic cancer, hepatocellular carcinoma, and breast cancer. The anti-CD44v6 single-chain variable fragment (scFv (CD44v6)) screened from the human phage antibody library was assembled into vesicles with an amphiphilic copolymer. Arsenite ions (As) were encapsulated in the core of vesicles to prepare vesicle nanoparticles (scFv-As-NPs). Experiments showed that the prepared arsenic nanovesicles had a better effect of inhibiting tumor growth and promoting tumor cell apoptosis, providing an efficient and safe platform for the delivery of mineral drug arsenic [68]. (−)-Epigallocatechin-3-gallate (EGCG) is a phenolic compound with rich content of tea polyphenols and good anti-tumor activity. Recent studies have shown that the combination of EGCG and metal ions can significantly improve drug activity and reduce metal toxicity, more importantly, it is also a good nano-drug carrier [69, 70]. Wei et al. combined the realgar with EGCG and prepared the nano-drug (EGCG-RNPs) by coprecipitation method. It was proved that EGCG as a carrier could significantly increase the uptake of HL-60 cells to realgar, which provided a new treatment for APL. In recent years, the great advantage of protein biomineralization to synthesize nanoparticles to enhance the synergistic effect of drugs has been reported in the literature. Macrophage membrane has good biocompatibility and can be used as a carrier for biomimetic nano preparations. During the treatment of atherosclerosis, Huang et al. [71] used macrophage membranes to wrap Fe_3_O_4_ to prepare liposome Fe_3_O_4_@M nano-drugs, which can be used for magnetic resonance targeted detection, which can better detect and treat the early stage of the disease stage. Bovine serum protein is also a common biomimetic nanomaterial. FeS_2_ nanoparticles synthesized by protein biomineralization have synergistic tumor therapeutic effects in vivo and in vitro photothermal photodynamic therapy and have no significant toxicity [72]. All these have proved that compared with traditional preparations (pills, powders, decoctions, etc.), modern pharmaceutical preparations can give full play to the characteristics of low-dose, low-toxicity, targeted transportation, and better play the role of mineral medicine effect.

### Carrier function of mineral medicine in disease diagnosis and treatment

#### Magnetic nanoparticles

**Fig. 8:**
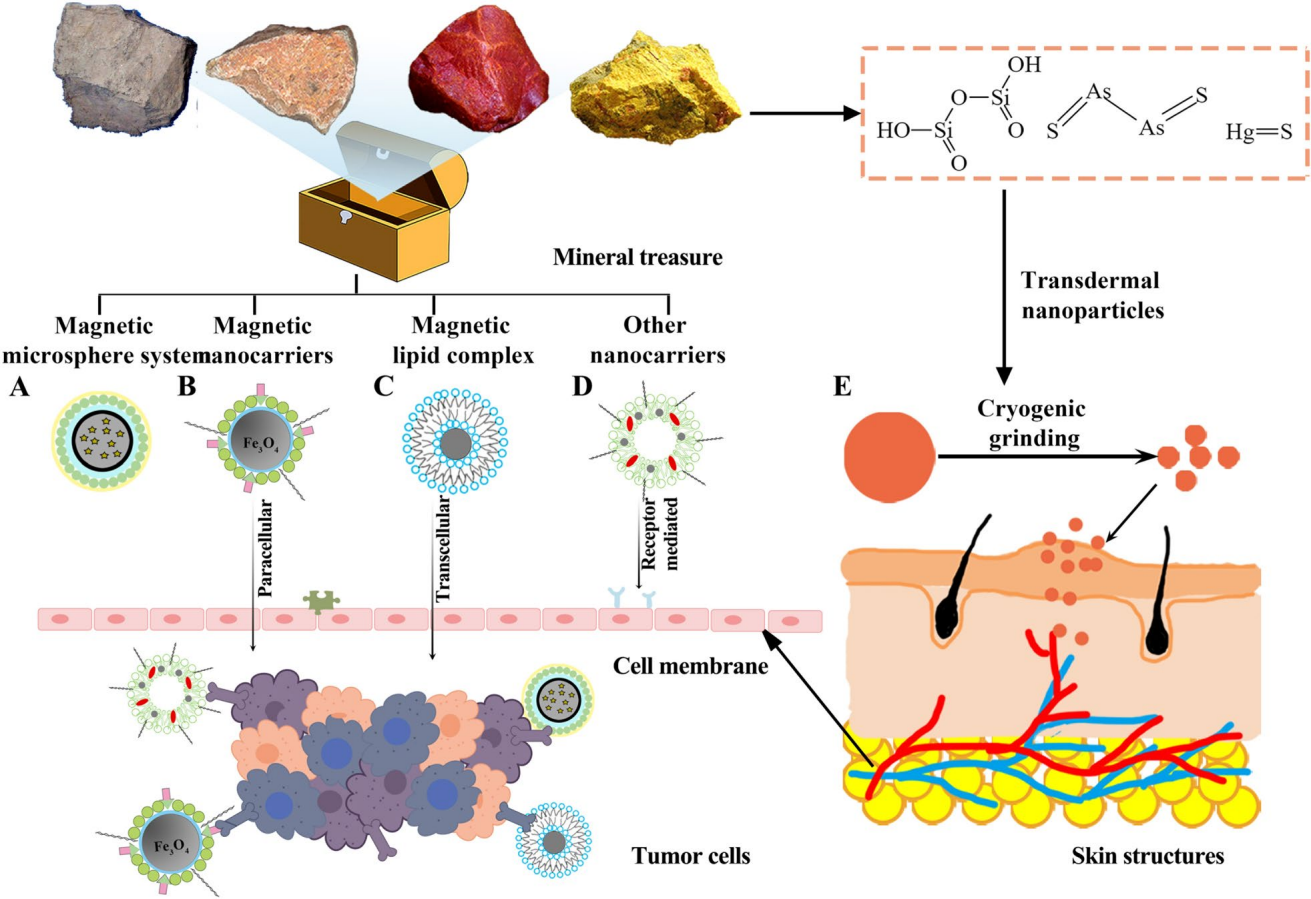
Schematic synthesis of mineral drug nanocarrier. The schematic diagram shows the application of magnetic nanoparticles, magnetic lipid complexes, magnetic microsphere systems, and percutaneous administration in mineral drug delivery systems. (The pictures in the figure from network sources have been allowed. Copyright^©^ 2014–2021 588ku.com. Copyright^©^ 2011–2021 ZHONGYIBAIKE.COM All rights reserved. Copyright^©^ 2019 www.tcmdoc.cn.)

With the growing trend of drug delivery system research, nanotechnology has become the focus of modern research owing to its target ability, longer circulation in vivo, and fewer side effects [73]. Among them, magnetic nanoparticles have many effects in cancer treatment for their good biocompatibility, quick magnetic response, and excellent fluorescence imaging. And it exhibits good application prospects in magnetic resonance imaging (MRI), magnetic fluid hyperthermia (MFH), and magnetic drug targeting (MDT) therapy. MRI is based on the fact that magnetic nanoparticles are coupled with fluorescent agents for clinical optical diagnosis. The report has shown that tumors can form damage or deactivation when the temperature reaches or exceeds 43 ℃. Therefore, the principle of MFH is to apply high temperatures to kill cancer cells [74]. As shown in Fig. 8B, magnetic nanoparticles (MNPs) can utilize a magnetic field to generate heat to achieve the purpose of thermotherapy for their excellent magnetism. However, it may also cause damage to normal and healthy cells probably owing to the lack of selectivity of hyperthermia. Drug targeting is the key to specific therapy, and magnetic nanoparticles can effectively accumulate in the target tissue and site when magnetic fields take effect. In addition, MNPs can also exert active targeting through selective through its targeting ligands binding to tumor over-expressed receptors [75]. When MRI, MFH, and MDT combine, showing effective diagnosis and treatment.

The general preparation methods of MNPs include coprecipitation and organic thermal decomposition. The coprecipitation method is prone to nano-agglomeration. Therefore, the precipitation hydrolytic alkali method, has relatively good stability and dispersion without high boiling point solvent and high temperature, suggesting a good substitute [76]. In magnetic nanoparticles, drugs can be coupled through covalent or non-covalent pathways. Non-covalent coupling refers to physical adsorption and entrapment, while covalent binding refers to the formation of covalent chemical bonds between MNPs and drug molecules. Covalent binding between drug loading and release is more difficult than non-covalent binding. Moreover, the residual catalyst in the binding process is also toxic to the physiological environment. When MNPs reach the designated site, it is released under-stimulation, including chemical and biochemical stimuli and external magnetic, light, and thermal stimuli.

Nanoparticles have always shown excellent efficacy in the treatment of cancer. However, there are some problems to be further studied, such as less targeted accumulation in vivo, short cycle time, ease to be eviscerated, and so on. Therefore, optimizing the physicochemical properties and modifying the surface of nanoparticles has become the focus of follow-up research [77]. The report showed that the target ligand, an amino-terminal fragment (ATF) peptide, binds to the urokinase plasminogen activator receptor. Targeted delivery of the hydrophilic drug gemcitabine (Gem) by coupling with magnetic iron oxide nanoparticles (IONPs) (Fig. 7A, B). Figure 7C, D showed that cell uptake was significantly increased in the ATF-IONP-Gem group after Prussian blue staining, about three times as much as in the non-targeted group. Figure 7E shows that in the IONPs-Gem group without ATF modification, the cell uptake of cancer cells was still quite low even at high concentrations, indicating that the modification of ATF enhanced the targeted therapy of drugs. In addition, Fig. 7F shows that the cytotoxicity experiment showed that the ATF-IONP-Gem group had a better level of cell survival than other groups, demonstrating that it reduced the cytotoxicity [78]. Folic acid-modified magnetic iron oxide bovine serum albumin (BSA) nanoparticles were also designed. At the same time, doxorubicin (DOX) was loaded to achieve the purpose of targeted therapy, and the key to targeted therapy was folate receptor overexpression cells in tumors [79]. Through the observation of transplanted tumor tissue sections, the results showed that the FA-DOX-BSA MNP hyperthermia group showed significant differences from MFH and MDT groups in an inflammatory response and cell necrosis. This indicates that targeting ligand coupling magnetic nanoparticles possess good antineoplastic activity. In addition, Lee et al. utilized silica magnetic nanoparticles to load drugs. Silica-mediated nanoparticles have stable chemical properties, low cytotoxicity, and large pore volume, which can provide a better platform to carry drugs and imaging agents [80].

#### Magnetic lipid complex

As an endogenous nano-carrier, liposome is composed of three major biological components: phospholipid bilayer, transmembrane proteins, and internal core. Liposomes have the following advantages: (1) high biocompatibility and low toxicity, (2) the ability to carry both water-soluble and water-insoluble drugs, (3) the highly modifiable surface, (4) the controllable size. MNPs exert multi-mode therapy in clinical treatment and become the focus of modern diagnosis and treatment [2]. Liposome and magnetic nanoparticles are utilized to be shell and core, respectively. This core–shell structure, also known as lipid magnetic nanoparticles (MLs), can effectively maintain the colloid stability of nanoparticles and prevent drug leakage (Fig. 8C). In the bilayer membrane of liposomes, the inner membrane is firmly adsorbed on the magnetic nanocomposites. While the outer membrane is relatively loose and easy to functionalize. For example, polyethylene glycol is utilized to perpetuate the cycling time in vivo. Otherwise, the modification of targeted ligands and fluorescent agents is to achieve drug targeting and bimodal imaging, respectively. Choi et al. reported that magnetic liposomes showed a strong anti-cancer effect and tumor targeting on colorectal cancer cells of CT26 mice under the action of an external magnetic realm [81]. Ketkar et al. achieved high hepatocyte targeting by designing lactose functionalized magnetic liposomes [82]. Soenen et al. reported that MLs can perform simultaneous MRI and fluorescence imaging because of the particularity of the bilayer of liposomes [83, 84]. Therefore, the organic combination of liposomes and magnetic nanoparticles is the optimization of a single delivery carrier among them. In addition, the research began to pay attention to some new types of liposomes, which not only have the advantages of liposomes but also have their unique properties. For example, pH-sensitive liposomes can be selectively released in weakly acidic tumor anaerobic areas [85].

#### Magnetic microsphere system

Microsphere refers to the dispersion system formed by drug dispersion in natural polymers and matrix polymers. Natural polymers comprise starch, albumin, and gel [86]. The matrix polymers of microspheres include polylactic acid, polylactic acid glycolic acid, and chitosan. Among them, PLGA has become the first choice of drug carriers for its high stability and entrapment efficiency [87]. Therefore, the microspheres show good biocompatibility, biodegradability, and sustained release. However, the clinical study indicates that restrictions of microsphere preparation have remained, such as low drug loading, low targeting, and poor stability [88].

Now, most studies have focused on strategies to improve drug loading and release. These strategies can be divided into four groups: (1) optimization of polymers: low molecular weight and hydrophobicity, (2) regulation of the external water phase PH, (3) increased polymer concentration, (4) emulsifier addition. Otherwise, Fig. 8A illustrates the application of magnetic nanoparticles (MNPs) to encapsulated microspheres is also gradually emerging. MNPs have excellent magnetic targeting and colloidal stability. Thus, the combination of magnetic nanoparticles and microspheres can play their respective advantages. And that provides a new direction for the clinical treatment of nanometer micro balloons. Srinivasan Ayyanaar and other researchers designed an associated system composed of iron oxide nanoparticles and poly (d,l-lactide-*co*-glycolic acid) to load curcumin [89]. MTT and cell colony formation experiments showed that the designed nano-system exhibited satisfactory cytotoxicity to cancer cells and could effectively inhibit cell colony-forming. Moreover, the morphological characterization experiments showed that the system also had good stability. In addition, the drug liberates tentative showed that the drug was targeted released in cancer cells under the action of the magnetic field. These experimental results show that the microspheres encapsulated by magnetic nanoparticles can better solve the problem of single microsphere preparation.

#### Other nanocarriers

Except for microspheres and liposomes, Fe_3_O_4_ NPs can also be designed as other preparations, including gels, vaccines, and aerosols. Nahid and other researchers designed multi-stimulus-responsive magnetic nano-hydrogels (MNHGs) to load doxorubicin hydrochloride [90]. The results of anti-cervical cancer experiments demonstrated that compared with single magnetic nanoparticles, MNHGs showed higher drug loading rate and cytotoxicity. In addition, owing to their superior pH, magnetic field, and thermal response, MNHGs show splendid targeting ability. Yi Zhao et al. focused on the potential of iron oxide nanoparticles to be adjuvants for cancer vaccines. In this research Fe_3_O_4_ NPs not only work as an antigen presentation system but also as immune enhancers for antigens. The results of immune response and anti-colon tumor experiments showed that the preparation induced a stronger immune response and improved the anti-tumor effect [91, 92]. Of note, Fe_3_O_4_ NPs owing to their superior magnetic targeting provide a new idea for the aerosol treatment of bronchogenic carcinoma. Mehdi Mohammadi et al. verified the targeted therapeutic effect of magnetic aerosol drug targeting through the Weibel symmetrical tree lung model [93].

Halloysite is a submicron hollow cylindrical aluminosilicate clay. Halloysite is a natural carrier with good biocompatibility and sustained-release properties, which can be used to transport a variety of drugs (Fig. 8D) [94–97]. Viviana et al. designed halloysite nanotubes to transport resveratrol. The system has shown an ideal encapsulation effect and an excellent ability to release the drug slowly, thus improving the efficacy of tumor therapy [98]. A novel co-delivery method of polysulfone mucopolysaccharides embedded in amine-functionalized halloysite nanotubes is reported in this paper. For this reason halloysite nanotubes are ideal materials with large pores that can absorb the mucus barrier and are sensitive to pH value. In addition, the delivery system can improve the stability of lactase and enhance the permeability of drug-entrapped mucosa [99]. This new microcapsule design provides a promising solution for the development of oral formulations.

### Discussion on transdermal drug delivery

Transdermal drug delivery is a kind of drug delivery modus, which makes the drug enter the blood circulation through the skin and mucous membrane. The way of treating diseases by skin medication has a long history of application in TCM preparation. For example, the medicinal paste in TCM is a representative and early transdermal delivery system. Its unique pattern of administration determines that transdermal drug delivery has the following characteristics: (1) The first-pass effect of the liver is avoided. (2) The degradation of drugs in the gastrointestinal tract was avoided. (3) It can not only play a role of systemic therapy but also act the part of local therapy. (4) It can make the drug hold a constant blood density, and reduce the number of drug administration. (5) The method of skin administration is safe and convenient. In the literature related, partial transdermal administration is favorable for the treatment of highly localized nubbles, such as breast cancer and cervical carcinoma [99–103]. However, the efficacy of percutaneous administration is often affected by the stability of microorganisms or enzymes on the skin surface and drug particles. This prompted researchers to seek new transdermal drug technology. For example, combined nanoparticles, liposomes, microspheres and another nano-sized particles with transdermal technology to design novel transdermal formulations can greatly improve the therapeutic effect. Toyoda et al. effectively delivered the nanogel containing antigen peptide to tumor cells through the skin by the cancer antigen inoculation test, which proved that the method of delivery antigen peptide via the skin is very beneficial to inhibit the growth of tumor [104]. The application of fresh techniques and methods provides a new research path for the transdermal drug delivery system. For example, realgar has been largely used since ancient times in the curing of various diseases, including snakebite, convulsions, and malaria. Modern research has also certificated that realgar has great potential in the treatment of various hematologic and solid tumors, such as breast cancer, granulocytic leukemia, skin cancer, etc. [105, 106]. Similarly, water-insolubility and toxicity have always been difficult problems limiting the clinical application of realgar. Here, Qi and his team used a transdermal delivery system for the first time to deliver realgar nanoparticles and evaluated their anticancer effects and toxicity in vivo (Fig. 8E). The result indicated that compared with intraperitoneal administration, transdermal delivery of realgar nanoparticles could significantly dwindle tumor volume, with higher efficacy and lower systemic toxicity [107]. At present, there are few studies on the transdermal delivery of mineral drugs. Traditional ointments involving mineral drugs have unique therapeutic advantages and long application history. However, there are still many problems to be solved in mineral drug transdermal preparations. The mechanisms of action of most mineral drugs and their interactions with diseases, the toxicity of mineral drugs, and the accumulation of heavy metals after transdermal administration are still unclear and need to be studied. Therefore, percutaneous administration of mineral drugs is a promising and challenging direction.

## Conclusions

Mineral medicines, as an integral part of the traditional medicinal culture, have been playing an important role for more than two millennia. Only 32.9% of mineral drug resources have been documented in the 2020 edition of the Chinese Pharmacopoeia [108], while mineral resources are widespread and have unique curative effects. Therefore, the development, research, and application of mineral drugs are still promising.

Mineral medicine, same as animal medicine and botanical drug, is an indispensable part of TCM. Mineral medicines have a lengthy history of application and reliable clinical efficacy. Experts in TCM of the past generations have summed up many valuable medicines' experiences and theories. Along with the advance of modern scientific knowledge, mineral medicines have also shown significant advantages in multiple malignant tumors therapy [23]. Mineral drugs commonly include heat-clearing mineral drugs (gypsum, mirabilite, realgar, etc.), hemostatic mineral drugs (ochre, alum, forged gypsum, etc.), sedative mineral drugs (cinnabar, lapis lazuli, magnetite), and insecticidal mineral drugs (cinnabar, calamine, etc.). For example, “Angong Niuhuang Pill” is an outstanding Chinese patent medicine, which mainly contains two mineral medicines of cinnabar (HgS) and realgar (As_4_S_4_). It has been popularly used for thousands of years to cure the central nervous process and cardiovascular diseases. Modern clinical treatment for brain trauma, hemorrhage, and coma has an excellent effect [109–113]. In addition, the coronavirus pneumonia disease that broke out in 2020 is raging all over the world, threatening the life of all mankind. Surprisingly, “Lianhua Qingwen capsule” and “Qingfei paidu decoction” have achieved very positive results in anti-novel coronavirus and life-saving. And these are well-known traditional Chinese medicine prescription preparations. In the above two kinds of TCM, there is an important mineral drug: gypsum. Gypsum is outstanding for its efficacy in clearing heat-fire and promoting fluid production. And doctors of TCM have commonly applied gypsum in exogenous fever, lung heat, asthma, cough, and other diseases. Therefore, it has broad prospects for an inheritance, especially in the exploration and forthputting of mineral drugs.

However, similar to the problems encountered in the study of all TCM components, the extensive pharmacological activity and clinical efficacy of mineral medicines are still to be studied. Besides, the pharmacological mechanism still needs a lot of exploration. In addition, the safety of mineral drugs is still the bottleneck in their development as a result that most mineral drugs contain a large number of heavy metals. For example, cinnabar is often used to treat neurological diseases [114–116]. And reports indicate that cinnabar and its main component HgS have low cytotoxicity. In in vitro experiments, the survival rate of cells in the environment of large-dose cinnabar and HgS is about 65%, higher than HgCl_2_ and MeHg. That means cinnabar has no significant effect on the intracellular mercury content [117]. However, studies have pointed out that even therapeutic doses of cinnabar have potential neurotoxicity [118]. Studies have pointed out that the cure and venom dosage of cinnabar have distinct pathways and targets in the cerebral cortex [114]. Moreover, the latent molecular mechanism of the pharmacodynamics and toxicological activities of cinnabar is still being explored. Some traditional mineral medicines in TCM are not used as medicines to play a therapeutic role in modern applications. Such as magnetite as a contrast agent component in the imaging of early atherosclerotic lesions [119]. This provides us with a very promising idea for the development of new pharmaceutical excipients, which embodies the advantages of the combination of TCM and accessories. Although there are some limitations in the methods of studying the pharmacological action and mechanism of a single mineral component. Considering that the mineral component is small and relatively simple, this research idea can also provide some references for the further study of mineral drugs [119].

At present, many works of literature have given an account of the therapeutic effects of the mineral drugs in a variety of malignant tumors, such as in the therapeutics of promyelocytic leukemia, gastric cancer, etc [120–124]. However, the safety issues such as heavy metals and hazardous elements in mineral drugs still limit the clinical use of mineral drugs. For example, the existing mineral preparations such as Daqi Li powder, Jiuhua ointment, and Half-sulfur pills on the market have shown therapeutic effects in removing blood stasis and swelling, repairing skin wounds. However, even at the dosage used, mineral medicines still unavoidably have certain hepatotoxicity and nephrotoxicity, as well as the accumulation of heavy metals. Therefore, it is very necessary for the development and utilization of mineral medicines to optimize mineral medicine preparations, improve the curative effects, and reduce toxicity by taking advantage of modern preparations.

New drug delivery systems and formulation technologies have established strategies for the study of mineral drugs in TCM, enabling many breakthroughs in the development and utilization of mineral drugs. The application of nanocarriers and biomaterials provides feasible opportunities for the construction of mineral drug nano-delivery platforms. Liposomes, exosomes, and polymer nanoparticles are widely used in nano-medicine for their superior biocompatibility. And they can be effectively designed as carrier materials for transporting mineral nanoparticles. It provides more possibilities for mineral drugs to play a therapeutic role. And they can be effectively designed as carrier materials for transporting mineral nanoparticles. It provides more possibilities for mineral drugs to play a therapeutic role. On the other hand, the unique properties and structures of mineral drugs have also shown great latent in the study of drug delivery carriers. Such as silicon dioxide coating which can protect insulin-loaded liposomes from degradation by digestive enzymes [16]. And another, calcium sulfate supports vancomycin nano-systems, which can significantly enhance their antibacterial properties [125].

So far, more studies on arsenic and iron preparations have been reported. The establishment of novel drug delivery systems greatly improves the drug loading and targeting of ATO, while the research on iron is mainly in MRI imaging ability and carrier research. Recently, many pharmacological activities of magnetic targeting systems have shown significant advantages, and iron minerals such as iron oxide have been further studied as drugs and carriers. In addition, in the design of mineral medicine preparations, due to the reduction in the amount of mineral medicine used, it is also possible to choose another medicine to match it. Under the synergistic treatment, it can better exert the efficacy of mineral medicine itself and reduce the dose toxicity, which is not only safer to use, but also effective even better.

In this review, we systematically describe the chemical constituents, pharmacological actions, and applications in delivery systems of mineral drugs of Chinese medicine, such as preparing multifunctional nanoparticles to deliver ATO to heighten the treatment of HCC. Despite the significant achievements in the research of mineral medicines delivery systems, there are still exist some challenges. The toxicity and safety of mineral drugs are still the focus of attention, and the more efficient and safe delivery matrix needs to be excavated. In addition, this review focuses on the research achievements of TCM mineral drugs in transport systems, including the curing of malignant burls and the progress in delivery vehicles. It is expected to overcome the defects of poor solubility, low bioavailability, and strong toxicity of TCM. Hopefully, under the reference of this article, it can help to tap more potential of mineral drugs in the research of drug delivery systems. Whether as medicaments or nano-delivery vehicles, we are all willing and eager to witness that.

## Data Availability

Not applicable.

## Abbreviations

AML: Acute myeloid leukemia
ANG: Angiopep-2
APL: Acute promyelocytic leukemia
ATF: Amino-terminal fragment
ATO: Arsenic trioxide
ATRA: All-trans retinoic acid
BBB: Blood–brain barrier
BHD: Bai-Hu decoction
BSA: Bovine serum albumin
BSM: Basilicum seed mucilage
CPX: Cephalexin
DCQD: Dachengqi decoction
DOX: Doxorubicin
EGCG: (−)-Epigallocatechin-3-gallate
FA: Folic acid
Gem: Gemcitabine
Gos: Gossypol
HBMEC: Human brain microvascular endothelial cells
HBV: Hepatitis B virus
HCC: Hepatocellular carcinoma
ICP-OES: Inductively coupled plasma optical emission spectrometry
IL-17: Interleukin-17
IL-1β: Interleukin-1β
IONPs: Iron oxide nanoparticles
iRGD: Internalizing RGD
LIP: Liposomes
LO2: Normal human hepatocytes
MAN: Mannose
MDT: Magnetic drug targeting
MFH: Magnetic fluid hyperthermia
MLs: Magnetoliposomes
M-LPMSN: Magnetic large-pore mesoporous silica nanoparticles
MNHGs: Magnetic nano-hydrogels
MNPs: Magnetic nanoparticles
MRI: Magnetic resonance imaging
MSN: Mesoporous silica nanoparticles
MTT: Methyl thiazolyl tetrazolium
NPs: Nanoparticles
PAA: Polyacrylic acid
PCM: Phase change material
PEG: Polyethylene glycol
PLC: Polyethylene glycol and lactobionic acid modified chitosan
PLGA: Poly(lactide-*co*-glycolide) acid
RA: Retinoic acid
RNPs: Realgar nanoparticles
scFv: Single chain variable fragment
TCM: Traditional Chinese medicine
TNF-α: Tumor necrosis factor-α
TNF: Tumor necrosis factor
TPP: Triphenylphosphine
